# Connectivity enhances resilience of marine forests after an extreme event

**DOI:** 10.1038/s41598-025-87449-y

**Published:** 2025-02-11

**Authors:** Sofie Vranken, Thomas Wernberg, Armin Scheben, Albert Pessarrodona, Jacqueline Batley, Melinda Ann Coleman

**Affiliations:** 1 UWA Oceans Institute & School of Biological Sciences, The University of Western Australia, 35 Stirling Highway, Crawley, WA 6009 Australia; 2 Phycology Research Group, Ghent University, Krijgslaan 281 S8, 9000 Gent, Belgium; 3 Institute of Marine Research, Nye Flødevigveien 20, 4817 His, Norway; 4 School of Biological Sciences, The University of Western Australia, 35 Stirling Highway, Crawley, WA 6009 Australia; 5 Simons Center for Quantitative Biology, Cold Spring Harbor Laboratory, Cold Spring Harbor, New York, NY 11724 USA; 6 New South Wales Fisheries, National Marine Science Centre, 2 Bay Drive, Coffs Harbour, NSW 2450 Australia

**Keywords:** Global change, Extreme climatic events, Kelp, Marine heatwave, ddRAD, Ecological genetics, Population genetics, Marine biology

## Abstract

The resilience of populations to extreme climatic events comprises the resistance to withstand and the ability to recover, which depends on factors such as remaining genetic diversity and population connectivity. In 2011, a MHW caused a 100 km range contraction of kelp (*Ecklonia radiata*) off Western Australia, but recently recovering kelp forests were discovered. To understand mechanisms of recovery and determine if recovering populations are survivors or immigrants, we used genotyping-by-sequencing to assess patterns of genetic diversity and connectivity. We found that two of the three recovering kelp forests (PG1 and 2) were likely survivors whereas a third smaller population (PGCr 1) was likely produced through re-colonisation from nearby surviving forests. Connectivity was high among populations and migration analysis identified one population (Horrocks) as the most important source for the recovering kelps. All recovering populations had higher neutral genetic diversity, and similar putative adaptive diversity to surrounding surviving populations, suggesting local adaptation. Our results elucidate how mixed processes can contribute to kelp forest resilience following MHWs but cryptic survival and maintenance of population connectivity is key to recovery.

## Introduction

Extreme climate events (ECEs), such as heatwaves, droughts, fires, and floods, are expected to increase in intensity and frequency under global change^1–5^. ECEs can result in ecological catastrophes ranging from degradation of genetic diversity, local extinction, altered ecosystem functioning and reconfiguration of entire communities^6,7^. Recovery after extreme events is often challenging and many ecosystems or species do not recover or take decades to return to pre-ECE levels^8^.

Resilience to extreme events is influenced by both the level of initial capability to persist through the disturbance and the capacity to recover after being disturbed^9^. Variation in resistance towards extreme events can arise from spatially variable abiotic conditions (e.g. temperature) or through differences in biotic components such as local adaptation, genetic diversity or species interactions^8,10,11^. Recovery after extreme events depends heavily on the genetic makeup of the surviving populations^12,13^ and the recolonisation and dispersal capacity influenced by the number of, and distance to, surviving habitat patches^14,15^. Understanding these dynamics is critical for predicting long-term resilience and future vulnerability of species and populations to stressors.

Marine heatwaves (MHWs) are ECEs where sea surface temperature (SST) is higher than the 90^th^ percentile of the climatological mean SST for a minimum of 5 consecutive days^16^. MHWs have been increasing in duration and intensity all over the world^2,17,18^ with devastating ecological^19^ and socioeconomic consequences^20^, ranging from coral bleaching^21,22^, collapse of seagrass meadows^23^, loss of seaweed forests all over the world^24–28^, to behavioural changes that influence prey-predator interactions in urchins or fish^29,30^, and extensive mortality of benthic marine invertebrates^31,32^. Among the most notable victims of global warming and MHW, is one of the most dominant marine foundation species of the southen hemisphere, the kelp *Ecklonia radiata*. Along the east, west and south coast of Australia and Oman, warming has been linked to extensive die-offs and local extinctions of entire forests^18,24,33,34^, disrupting entire ecosystems and functions. For instance, one of the most severe MHWs ever recorded occurred off Western Australia during the Austral summer of 2010–2011. This event resulted in temperature anomalies up to + 5 °C for several weeks, spread over 12° latitude^35^ and leading to catastrophic impacts on benthic communities^21,23,36,37^, including kelp forests (*Ecklonia radiata*)^24,38^. Approximately 2,300 km^2^ of West Australian kelp forests were impacted, including a ~ 100 km range contraction at the warm range edge where kelp forests were replaced by algal turfs, small filamentous and foliose seaweeds tightly packed with sediments^24,34^. Kelp recovery was suppressed by a background of warm temperatures^11^ and an influx of tropical herbivores^24,39^. Despite the climate returning to pre-heatwave conditions in subsequent years^40^, annual diving surveys revealed a lack of recovery. In 2020 (10 years later) however, a few adult kelps were discovered in crevices on annually monitored reefs (PG crevices 1–3)^41^ and patches of kelp on newly monitored reefs (PG forest 1 and 2) situated at the northern edge within the area of range contraction. ranging from coral bleachinghelp to elucidate mechanisms of loss and predict future resilience of this ecologicallyimportant species, as well as develop conservation and management strategies^42,43^. 

When extreme events lead to local extinction and range contraction as was seen for these kelp forests, recovery is only possible through recolonisation from surviving surrounding populations e.g.^14^. Moreover, recolonisation after extinction is often restricted to the settlement of only a limited subset of founding individuals, creating reduced genetic diversity and genetic bottlenecks^44^, which can cause inbreeding depression and decrease signals of adaptation^45^, and negatively impact the fitness and resilience of the recovering population. In the same way, recolonisation after extinction can increase genetic differentiation between recolonising populations, particularly when population growth after colonisation is limited^46^. Generally, these founder effects are expected to be strong under low population connectivity and when recolonisation is established by only a few colonists from a limited number of source populations (the propagule model sensu Slatkin)^15,47^. In contrast, when recolonisation is established under high population connectivity and from multiple source populations, founder effects are expected to be limited or even absent (migrant model sensu Slatkin). Given the ecological and economic importance of *Ecklonia* forests^48^, understanding the mechanisms underlying resilience, recovery, and recolonisation after extreme events is paramount. Hence, to understand the recovery process and assess whether the newly discovered kelps are survivors or new immigrants, we assessed patterns of genetic diversity and population connectivity using genotyping-by-sequencing. Specifically, we tested the following hypotheses: (1) genetic diversity is low in newly discovered kelp forests due to founder effects, (2) newly discovered kelp populations are new immigrants coming from and showing the highest genetic similarity to the closest surviving populations, and (3) show similar signals of genetic adaptation to environmental variables, such as higher temperature, as their source populations.

## Methods

### Study species and sampling

*Ecklonia radiata* is one of the most dominant kelps in the southern hemisphere, where it occurs from the shallow subtidal up to 40m (rarely 80 m)^48^. It often forms monospecific forests, underpinning biodiversity and food webs and providing both ecological and socio-economical values^48^. It is considered one of the most warm tolerant kelp species globally, with a maximum temperature of 23 °C for sporophytes and 28 °C for gametophytes^48^. Despite being non-buoyant, its disjunct but extensive present (and historical) distribution (e.g. South Africa, Oman, Australasia) suggest a high ability to disperse and adapt to local conditions. It has a typical Laminarian lifecycle where a free-living diploid and macroscopic sporophyte (up to 2m) produce zoospores that develop into haploid, microscopic gametophytes (< 1 mm). Gametophytes produce eggs and sperm, which after sexual reproduction, produce sporophytes again^48^. Alternatively, they can delay development and form a bank of microscopic forms that persist during unfavourable conditions^49–51^. Asexual reproduction can occur in *E. radiata*, yet only in the distinctive ‘*brevipes’* morphotype endemic to Hamelin Bay^52^, which is not included in this study. For more background information on E. radiata, we refer the reader to a comprehensive review by^48^.

**Fig. 1:**
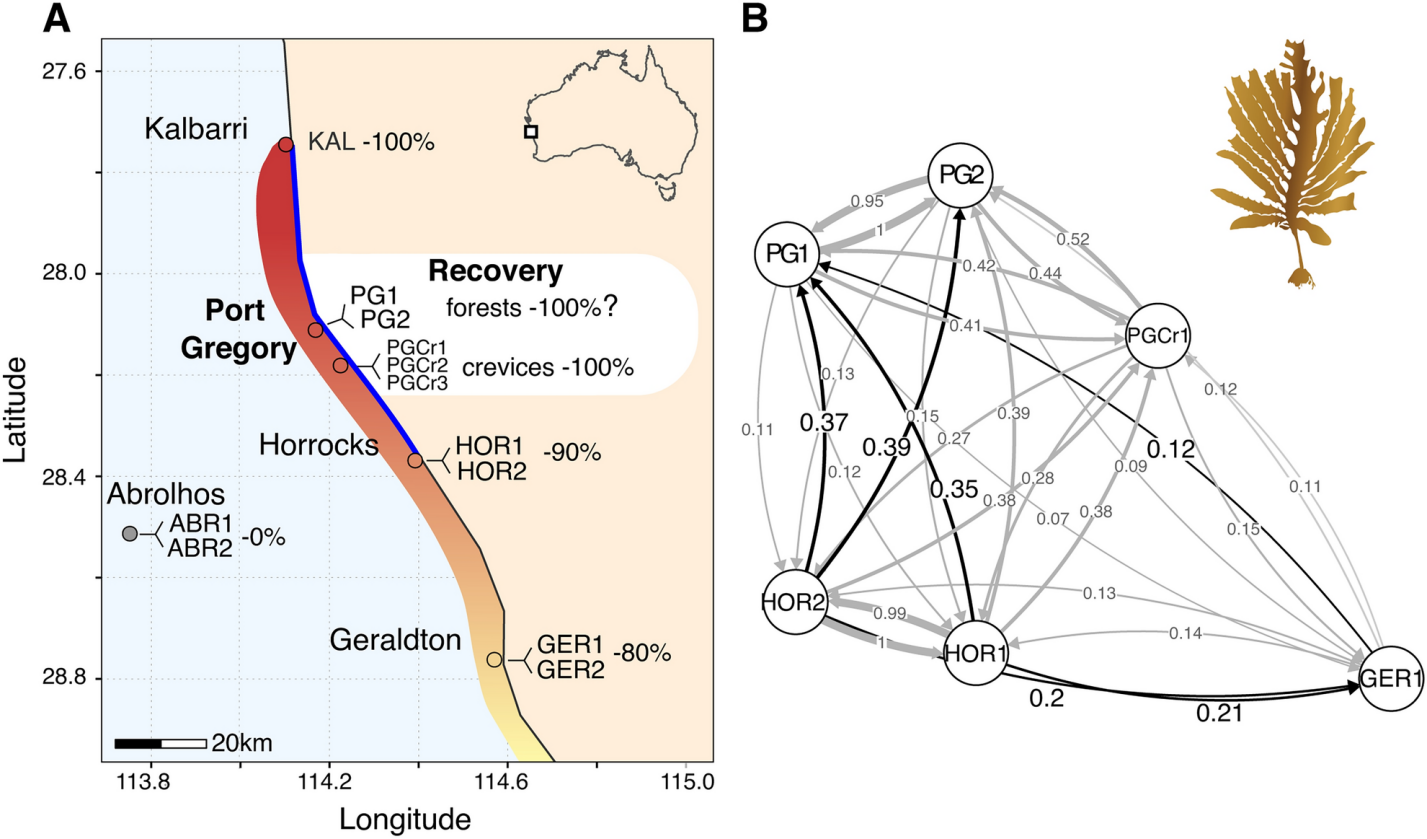
(**A**) Sampling location and sites for kelp (*Ecklonia radiata*) and the estimated percentage of forest loss after the 2011 marine heatwave (Wernberg et al. 2016). Bright blue indicates the estimated extent of range contraction. Red to yellow shading indicates the impact of the marine heatwave. (**B**) Bidirectional relative migration rates (m_R_) calculated with divMigrate^58^ and based on N_M_, using the main dataset (n = 6133 SNPs) to identiy source-sink dynamics. Open circles represent populations and are positioned according to relative migration rates; the stronger the migration rates between populations, the closer populations are positioned. Connecting vectors indicate migration directions and are weighted according to the relative migration rate (thin vectors indicate a low migration rate, thick vectors indicate a high rate). Significant asymmetric migration rates indicating source-sink dynamics are indicated in black. PGCr2-3 were excluded because of low sampling numbers (< 10). See Table 1 for abbreviation of sampling sites.

In 2018 and 2019, adult sporophytes were collected from healthy kelp forests from locations that were impacted by the 2011 MHW or occurring nearby (Fig. 1A, Fig. S1). These sites were (1) The Houtman Abrolhos Islands (ABR) where apparent little or no impact was reported, even if little quantitative data exist (pers. comm T. Wernberg), (2) Geraldton (GER) which suffered ~ 80% loss of coverage, (3) Horrocks (HOR) which suffered ~ 90% loss of coverage, and (4) Port Gregory (PG) which was thought to have suffered 100% loss of coverage with no kelps observed until recently (Fig. 1A). Within each location, we sampled two sites 1–4.5 km apart depending on rocky reef availability. At each site, 30 individuals were haphazardly collected by SCUBA at a depth of ~ 8.5m, with individuals being at least 1 m apart. Additionally, in PG, three additional reefs were sampled (PGCr) (Fig. 1A), where currently no healthy kelp forests are present but where scattered individuals were recently found in crevices and under overhangs^41^. When a kelp individual was found in a crevice, a small tissue sample was taken for DNA extraction from a lateral (digit). Drift sporophyte material has driven recolonisation of multiple kelps species with positive buoyancy^53–55^. Even though *E. radiata* is not buoyant*, E. radiata* plants can disperse long distances (10-100s km)^56,57^ with free-floating sporophytes often drifting over reefs (authors’ personal observation). Any drifting kelp individuals (i.e. non-attached and free-floating) encountered during these surveys were, therefore, collected and genotyped to determine their origin and assess if drift could be a possible pathway for recolonisation. For all collected samples (individuals, laterals, drift), clean and healthy-looking tissue was snap-frozen and stored at -80 °C until further processing. Lastly, we also genotyped dried material from 30 individuals collected prior to the heatwave (2006) from a fifth location, Kalbarri (KAL), the historic range edge which suffered a 100% loss of coverage and for which no recovery has been reported so far (Fig. 1A).

**Table 1:** Genetic diversity metrics for kelp populations (N > 10) surrounding Port Gregory, Western Australia.

Location	Sampling site	N	*N*_P_	% Loci	*H*_O_	*H*_E_	π	*F*_IS_ ± SE
Port Gregory forests	PG1	18	14	61.770	0.169	0.169	0.174	0.051 ± 0.016
	PG2	20	34	63.482	0.170	0.168	0.173	0.011 ± 0.016
Port Gregory crevices	PGCr1	10	8	55.634	0.166	0.161	0.171	0.013 ± 0.011
Horrocks	HOR1	20	10	59.448	0.148	0.149	0.154	0.041 ± 0.017
	HOR2	20	25	58.378	0.149	0.149	0.154	0.019 ± 0.017
Geraldton	GER1	20	138	57.210	0.168	0.158	0.163	− 0.000 ± 0.022

Number of individuals successfully genotyped (N), number of private alleles (*N*_P_), percentage of polymorphic loci (% Loci), observed heterozygosity (*H*_O_), Expected heterozygosity (*H*_E_), nucleotide diversity (π), and inbreeding coefficient (*F*_IS_).

### DNA extraction, SNP calling and SNP filtering

Fifty milligrams of frozen or dried material was ground and processed for DNA extraction by using the DNeasy plant DNA & Pro clean up kit (Qiagen, Hilden, Germany). A maximum of 20 individuals per sampling site were processed for DNA extraction and library preparation, except for the historical dried material from Kalbarri, where we extracted DNA from 30 individuals but of which only six individuals passed quality control for further library preparation. DNA quality control and ddRAD library preparation were performed as in Vranken, et al.^57^. Final libraries were sequenced on an Illumina Hiseq Xten at the Kinghorn Centre for Clinical Genomics’ Sequencing Facility (Darlinghurst, NSW, Australia). The Illumina base calls were converted to the fastq format and processed as in Vranken, et al.^57^ for de novo SNP calling using Stacks (v2.5)^59^. A minimum distance of three nucleotides was chosen to identify a stack (-m) and a maximum distance of three nucleotides was permitted between stacks in a locus (-M). A total of three mismatches were allowed between orthologous loci of different individuals when building the catalog (-n). These Stacks parameters were selected using the R80 method from Paris, et al.^60^, which was developed to optimize Stacks parameters by maximizing the number polymorphic loci repeatedly assembled across 80% of the population. Because the quality of the SNP calling of the dried Kalbarri samples was relatively low compared to the snap frozen material, two datasets were created; the main dataset excluding Kalbarri samples with a high number of SNPs retained after filtering, and a second dataset including Kalbarri samples but with a lower number of SNPs. To retain only high-quality SNPs in the main dataset, vcftools v0.1.15^61^ was used to first remove individuals with over 75% of missing data and remove all indels and multiallelic sites with requirements of a minimum and maximum depth of coverage of 3 and 20. Maximum missingness was limited to 20% per site and a minimum allele frequency (maf) of 0.03 was applied. Only SNPs that were present at six sampling sites and in at least 80% of the individuals within those sampling sites were included^60^. Further, loci not in Hardy–Weinberg equilibrium within more than 70% of the sampling sites (crevice sampling sites considered as one population) (*p* < 0.001) were removed using the script filter_hwe_by_pop.pl from the dDocent pipeline^62^ (https://github.com/jpuritz/dDocent/blob/master/scripts/filter_hwe_by_pop.pl). To reduce the probability of linked loci in the final dataset, only one SNP per RADtag was retained by thinning with vcftools. Lastly, individuals with more than 55% of missing data were removed with a custom Bash/R script, leaving a final set of 6133 SNPs and 155 individuals from 8 sampling sites (all individuals from GER2 were removed due to high missingness), for the main dataset. Depth of coverage per SNP varied between 6 × and 14x (mean 10x), and mean missingness per individual was 24%. For the dataset that included old Kalbarri samples, similar filtering was used except maximum missingness was more strict and limited to 10% per site and a maf of 0.02 was applied. This resulted in a dataset with 663 SNPs and 175 individuals from 10 sampling locations. Depth of coverage per SNP varied between 7 × and 13x (mean 11x), and mean missingness per individual was 11%.

### Population structure and colonisation and migration pathways

To characterise overall genetic structure, two clustering methods were applied within the R statistical environment; (1) Discriminant Analysis of Principle Components (DAPC), using *Adegenet*^63^, and (2) sparse nonnegative matrix factorization (sNMF) using *LEA*^64^. The best fitting number of groups (K), with K 1–10, was defined through minimal cross entropy (CE) and the Bayesian information Criterion (BIC)^64,65^. The number of principal components that was retained for DAPC was validated through cross validation on a training set including 90% of the individuals. Clustering was performed for both the main dataset (6133 SNPs) and the dataset including Kalbarri samples (663 SNPs). As preliminary analysis indicated that the Abrolhos sites were highly differentiated from the mainland sites and showed negligible admixture with mainland sites (Supplementary Fig. S2, Supplementary Fig. S5), the Abrolhos sites were excluded in the clustering, migration and assignment analysis to provide greater clarity among mainland sites. To further infer population connectivity among populations and identify populations that act as significant sources or sinks^66^, directional relative migration rates (m_R_) were calculated, using the main dataset (6133 SNPs) and the divMigrate function within the package *diveRsity*^67^. Here, the relative migration rate is estimated by calculating the genetic differentiation among a hypothetical pool of migrants for a given pair of populations and subsequently using the directional genetic differentiation to assess the relative levels of migration between the two given populations^58^. Relative migration rates were calculated based on all genetic differentiation metrics included (Jost’s D, GST, Nm) and ninety-five percent confidence intervals were calculated with 9,999 bootstrap iterations to identify significant net migration directions, i.e. source-sink dynamics. Only sites with more than 10 sampled individuals were included.

To test the hypothesis that newly discovered forests recolonised from nearby surrounding populations, two assignment analyses were performed. First, to infer the recolonisation pathways of the kelps in the PG crevices only, a genetic assignment of the kelps in PG crevices to potential origin populations was performed using the main dataset (6133 SNPs). Potential populations of origin were here Geraldton, Horrocks and PG forests. Next, to infer the recolonisation pathway of all kelps found in PG (forests and crevices), the dataset including Kalbarri samples (663 SNPs) was used, and all samples from PG (forests and crevices) were assigned to potential origin populations (here: Geraldton, Horrocks or Kalbarri). Membership probabilities were calculated with a naïve Bayes model in *assignPOP*^68^. Individuals were only assigned to a genetic cluster when the assignment probability exceeded 0.95. The analyses were cross-validated with Monte Carlo tests to assess self-assignment within each possible origin population.

To test if the newly discovered kelps are genetically different from the surrounding sampling sites and the historical Kalbarri samples, pairwise *F*_ST_ was calculated with the main dataset (6133 SNPs) and the dataset including Kalbarri samples (663 SNPs). Pairwise *F*_ST_ values between sampling sites were tested for Bonferroni corrected significance (*p* < 0.05) with 99 999 permutations using *Stampp*^69^. Mantel tests in *Adegenet* were used to test for associations between linearized *F*_ST_ (*F*_ST_/1-*F*_ST_) and geographic distance between the coastal sampling sites.

### Diversity metrics

To test the hypothesis that newly discovered populations are characterised by reduced genetic diversity caused by founder events, genetic diversity was estimated for each sampling site including more than 10 individuals (omitting PGCr2-3 and drift samples). Mean nucleotide diversity (pi), percentage of polymorphic loci (% Loci), number of private alleles (*N*_P_), mean expected heterozygosity (*H*_E_), and mean observed heterozygosity (*H*_O_), and inbreeding coefficient (*F*_*I*S_) were calculated in Stacks using all SNPs (variant and fixed) as recommended by Schmidt, et al.^70^. Differences in *H*_E_ among populations were tested with pairwise Wilcoxon tests corrected for multiple comparisons using the Bonferroni method. All diverisity metrics were calulated with the main dataset (6133 SNPs).

### Trends of putative temperature adaptation

To test the hypothesis that the newly discovered kelps have similar patterns of putative adaptation to temperature stress as the source populations they recolonised from, we applied a DAPC clustering analysis and compared allele frequencies of putative temperature linked loci among sampling sites. Historical samples from Kalbarri were included to explore how the recovering populations differ in trends of adaptation compared to extirpated populations. Candidate loci associated with temperature have been characterised for populations along the West coast of Australia, including some 80 km away from the ones studied here, in Vranken, et al.^57^ by using latent factor mixed model (lfmm) and partial canonical redundancy (RDA) analysis. To examine patterns of putative adaptation, all rad-tags including these putative temperature-linked-loci (174) were identified and a DAPC analysis was performed as explained above. Allele frequencies were calculated per sampling site using *PopGenReport* v3.0.4^71^ and the newly discovered kelps (PG1-2, PGCr 1–3) were visually compared to sampling sites poleward of Geraldton taken from Vranken, et al.^57^ (Fig. S8B). Annotations for the putative temperature-linked-loci were taken from Vranken, et al.^57^.

## Results

### Population structure and colonisation and migration pathways

**Fig. 2:**
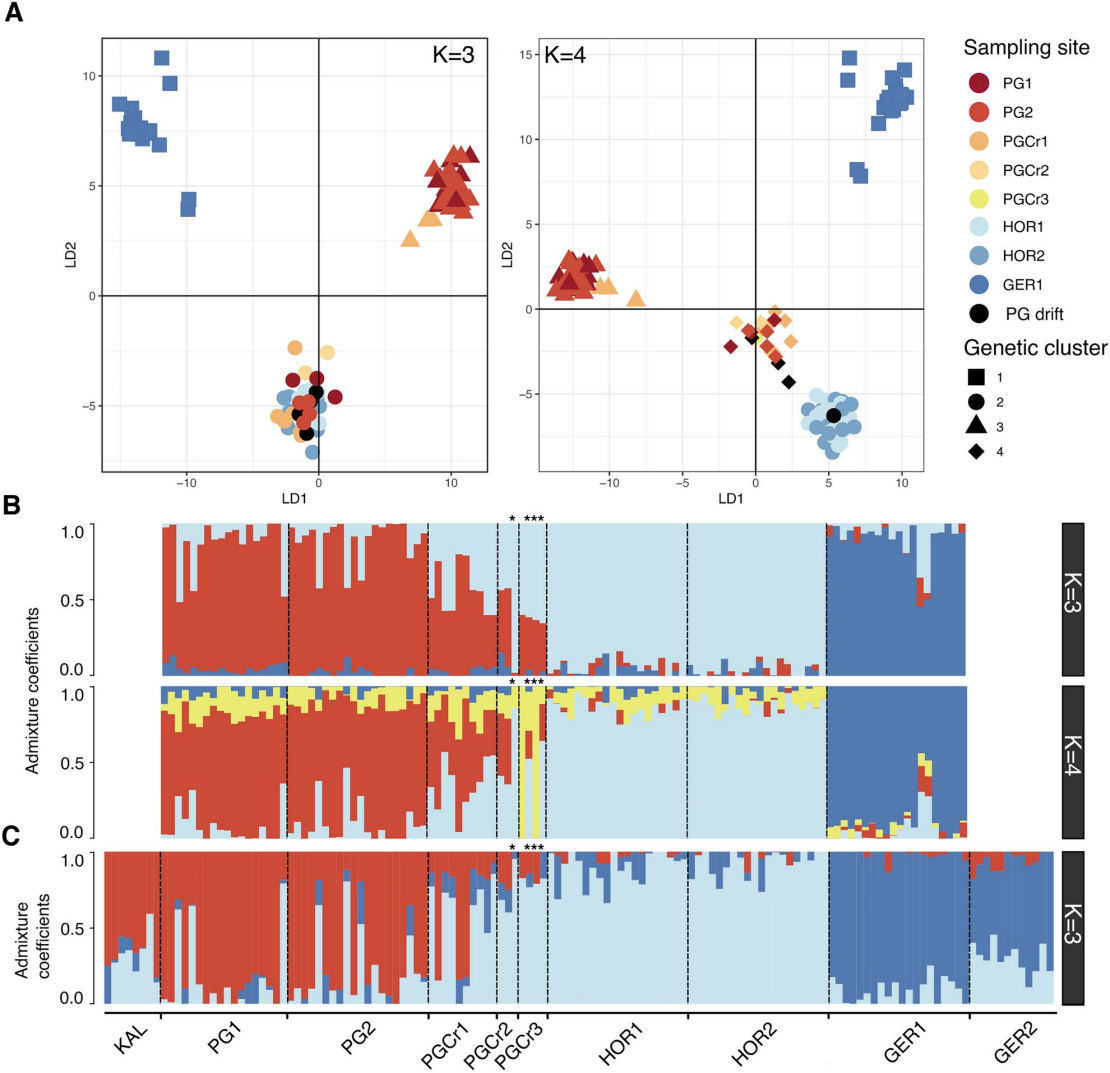
DAPC and sNMF clustering for *Ecklonia radiata* using the main dataset (6133 SNPs) (**A**,**B**). (**A**) DAPC scatter plot for K = 3 and K = 4, only the first 2 discriminant functions are shown, shape of data points refers to the genetic cluster and colour refers to sampling site, drift individuals collected at PGCr2 or PGCr3 are coloured black. (**B**) sNMF admixture coefficients for K = 3 and K = 4 per sampling site. (**C**) sNMF admixture coefficients for K = 3 per sampling site using the dataset including historical Kalbarri samples (663 SNPs). For the sNMF figures, every bar represents one sampled individual with every colour representing the membership proportion of each cluster, dashed black lines separate sampling sites, drift specimens are indicated with *.

For the main dataset (6133 SNPs), the most likely K was identified as K = 4 by DAPC and K = 3 by sNMF (Figure S3). Only the optimal K is discussed but K = 3 and K = 4 are plotted for comparative reasons. For K = 4, DAPC analysis clustered three individuals (30%) from one of the PG crevices sites (PGCr1) together with almost all individuals from the PG forests (PG1-2). The remaining individuals from PG crevices (PGCr1-3) grouped together with seven individuals from PG forests (PG1-2) and three drift individuals collected at PG crevices. One drift individual collected at PG crevices (PGCr2) clustered with individuals from Horrocks (HOR1-2), suggesting long distance dispersal by sporophyte drift from Horrocks to PG crevices (> 25km) (Fig. 2A). Individuals from Geraldton (GER1) formed their own cluster (Fig. 2A). For sNMF K = 3, most individuals from PG crevices (PGCr1-3) showed almost equal admixture with Horrocks (HOR1-2) and PG forests (PG1-2), except for one drift individual from PG crevices (PGCr2) that was fully assigned to the Horrocks cluster (Fig. 2B). PG forests (PG1-2) showed limited admixture with Horrocks (HOR1-2) except for six individuals that showed relatively more admixture (30–50%), possibly indicating early generation migrants (Fig. 2B). There was admixture between Horrocks (HOR1-2) and all other sampled sites (Fig. 2B).

For the smaller dataset (663 SNPs) that included historical Kalbarri samples prior to the heatwave, K = 3 was identified as the most likely K by both DAPC and sNMF clustering (Fig. S4). For K = 3, DAPC clustered individuals from Kalbarri (KAL) with PG forests (PG1-2) and three individuals from PG crevices (PGCr2), indicating high resemblance between historical Kalbarri (KAL), PG forests (PG1-2), and a few PG crevice samples (PGCr1) (Figure S4). The remaining individuals from PG forests (n = 6) and PG crevices (PGCr1-3), including drift samples, clustered together with Horrocks (HOR1-2). Geraldton (GER1-2) clustered apart (Fig. S4). sNMF analysis for the Kalbarri dataset, indicated admixture between the historical Kalbarri (KAL) samples and PG forests (PG1-2) and, a smaller amount of admixture with Horrocks (HOR1-2) (Fig. 2C). Six individuals from PG forests showed ~ 50% admixture with Horrocks, potentially indicating recent migration. PG crevices were of mixed origin and showed admixture with the PG forests – Kalbarri cluster and Horrocks (Fig. 2C). In general, there was substantial admixture among adjacent sites (Fig. 2C).

Directional migration patterns calculated with the main dataset (6133 SNPs) were similar across the three diversity metrics used (Jost’s D, G_ST_ and N_M_), except the magnitude of the relative migration rates (m_R_) were higher for Jost’s D. We therefore only show results for one of these metrics (N_M_; Fig. 1B). There were very high relative migration rates (m_R_ > 0.95) between pairs of sampling sites within PG (PG1-2), but also between PG crevices and PG forests (m_R_ > 0.41) (Fig. 1B). All PG sites (forests and crevices) showed migration with all sites from the other locations (Geraldton, Horrocks), with relative migration rates between PG—Horrocks (m_R_ = 0.13–0.39) much higher than between PG—Geraldton (m_R_ = 0.07–0.15) (Fig. 1B). Bootstrapping revealed significant northward net migrations from Horrocks (HOR1-2) and Geraldton (GER1) towards PG forests (PG1-2), identifying Horrocks (HOR1-2) and Geraldton (GER1) as significant source populations for PG forests (PG1-2) (Fig. 1B). Horrocks (HOR1-2) also had the highest relative migration rate (m_R_ = 0.35–0.39), making it the most important source of the recovering populations in PG forests (Fig. 1B).

**Fig. 3:**
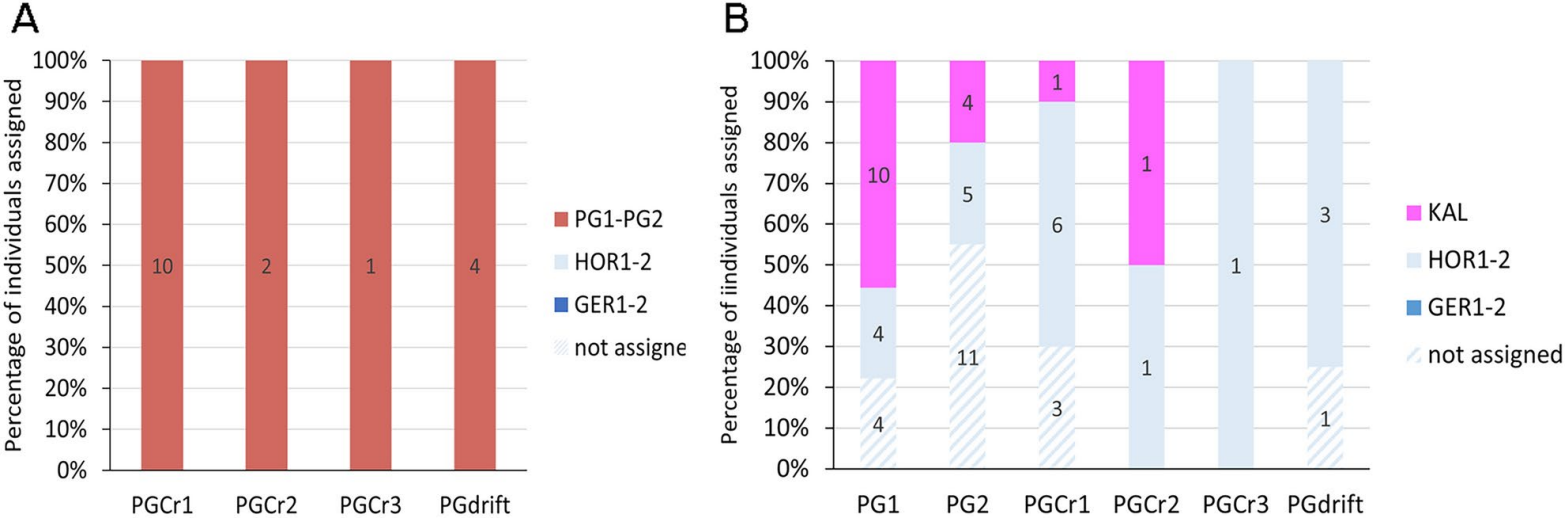
The percentage of samples from Port Gregory successfully assigned to surrounding genetic clusters using *assignPOP*. (**A**) individuals sampled at Port Gregory crevices (PGCr1-3) assigned to surrounding genetic clusters (PG1-2, HOR1-2, GER1) using the main dataset (SNPs = 6133). (**B**) individuals sampled at Port Gregory crevices (PGCr1-3) and Port Gregory forests (PG1-2) assigned to surrounding genetic clusters (KAL, HOR1-2, GER1-2) using the dataset including historical Kalbarri samples (663 SNPs). PG drift includes all drift samples collected at PGCr1-3. Samples were considered successfully assigned when assignment probability > 95%, see Supplementary Tables S1 and S2 for the posterior assignment probabilities per individual. Numbers within bars refer to exact number of individuals assigned.

Posterior assignment of individuals from the PG crevices (PGCr1-3) using the main dataset (6133 SNPs), assigned all individuals, including drift samples, to the PG forest cluster (PG1-2) (Fig. 3A, Supplementary Table S1). Mean self-assignment rates were 100% for all possible origin populations (PG1-2, HOR1-2, GER1), indicating clear delineation among these genetic clusters in the model applied (Supplementary Table S2). However, these results do not align with the DAPC (K = 4) and snmf (K = 4) clustering above, where at least one crevice individual is fully grouped with the Horrocks cluster (Fig. 2A,B).

Notably, posterior assignment of all PG individuals (crevices; PGCr1-3 and forests; PG1-2) using the Kalbarri dataset (663 SNPs) assigned 11 crevice individuals (including drift) to the Horrocks cluster (HOR1-2) and two to the Kalbarri cluster (KAL) (Fig. 3B, Supplementary Table S3). This is inconsistent with the assignment analyses above (Fig. 3A), but consistent with the DAPC and snmf clustering (Fig. 2C, Supplementary Fig. S4B) which groups some of the crevice individuals with the PG forests (PG1-2) and Kalbarri (KAL) individuals, and other crevice individuals together with all individuals from Horrocks (HOR1-2) and a few individuals from PG forests (PG1-2) (Fig. 2C, Supplementary Fig. S4B). Using the Kalbarri dataset, 14 individuals from PG forests (PG1-2) were assigned to the historical Kalbarri cluster (KAL) and nine to the Horrocks cluster (HOR1-2), congruent with the DAPC and snmf analysis (Fig. 2C, Supplementary Fig. S4B). Together this suggests possible survival through the heatwave of some PG individuals, but also recolonisation by a few individuals from Horrocks afterwards. Nineteen individuals indicated membership probabilities < 0.95 and were not assigned (Supplementary Table S3). Mean self-assignment rates varied from 77% (KAL) to 100% (PG1), indicating that there is some uncertainty around delineation among these genetic clusters in the model applied, especially for the Kalbarri population (KAL) (Supplementary Table S4).

**Fig. 4:**
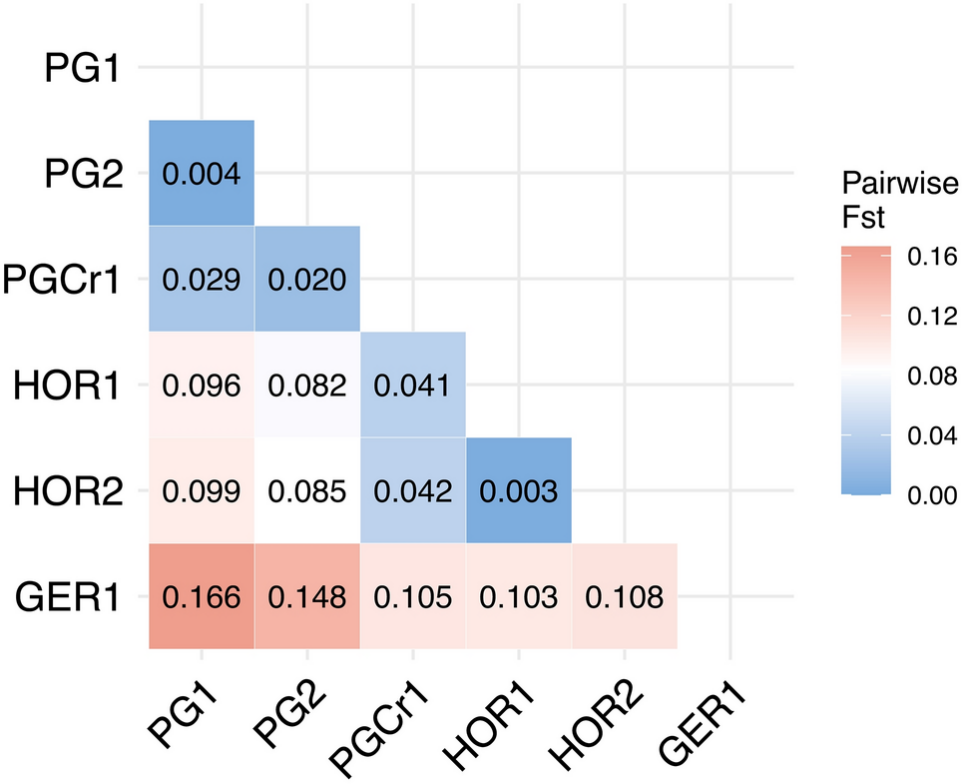
Pairwise *F*_ST_ estimates among sampling sites using the main dataset (6133 SNPs). All values were significantly different from zero (*p* < 0.05) and sampling sites are ordered from north to south.

Using the main dataset (6133 SNPs), pairwise *F*_ST_ analysis indicated small but significant genetic differences (*p* < 0.05) among all sites within PG, ranging from 0.0004 (PG1-PG2) to 0.029 (PG1-PGCr1) (Fig. 4). All sites within PG showed significant differentiation (*p* < 0.05) from sites from other locations, ranging from *F*_ST_ = 0.166 (PG1-GER1) to *F*_ST_ = 0.041 (HOR1-PGCr1) (Fig. 4), with significant isolation-by-distance (R = 0.9, *p* = 0.002). Genetic diversity metrics and pairwise *F*_ST_ values for the main dataset are also calculated including the Abrolhos and per genetic cluster (Figure S5, Supplementary Fig. S4).

When using the dataset including historical Kalbarri samples (663 SNPs), pairwise *F*_ST_ analysis indicated no genetic difference between the PG forests (PG1-2) and the historical Kalbarri population (KAL) (PG1-KAL: *F*_ST_ = 0.024, PG2-KAL: 0.040, *p* = 1), but a moderate significant difference between the PG crevices and Kalbarri (*F*_ST_ = 0.204, *p* < 0.01) (Figure S7), suggesting possible survival of the PG forests through the MHW, either as sporophytes or as microscopic gametophytes, and more recent recolonisation in PG crevices.

### Diversity metrics

Expected heterozygosity was higher for the PG forest (PG1-2: *H*_E_ = 0.169–0.168) and PG crevices sites (PGCr1: *H*_E_ = 0.161) than for other coastal sites (Table 1, pairwise Wilcoxon test *p* < 0.01, Supplementary Table S5). Nucleotide diversity followed a similar trend (PG1-2: π = 0.174–0173, PGCr1: π = 0.171) but the percentage of polymorphic loci were similar (Table 1). The inbreeding coefficients were similar for all sampling sites, indicating significant but overall limited levels of inbreeding. We observed private alleles for every genetic cluster with the least private alleles for the crevice site (PGCr1: *N*_P_ = 8), PG forests (PG1-2: *N*_P_ = 14–34) and Horrocks (HOR1-2: *N*_P_ = 10–25) (Table 1). Note that diversity metrics of the Crevice site should be interpreted with caution due to the small sample (N = 10) compared to the other sampling sites (N = 18–20). See Supplementary Table S6 for diversity metrics per genetic cluster.

### Trends of putative temperature adaptation

**Fig. 5:**
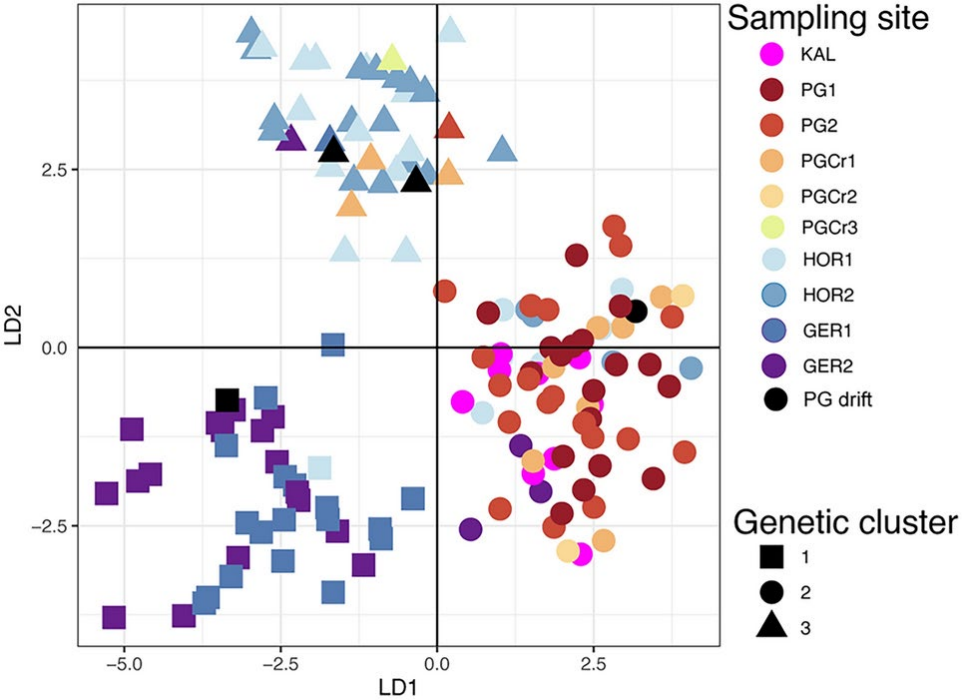
DAPC cluster analysis for putative temperature-linked loci (174 SNPs) for all sampling sites, only the first 2 discriminant functions are shown, shape of data points refers to the genetic cluster they belong to and colour refers to sampling site, drift individuals collected at PG crevices (PGCr1-3) are coloured black.

Individuals from PG forests and Crevices (PG1-2, PGCr1-3) showed similar trends in allele frequencies for SNPs putatively linked to temperature as the surrounding coastal populations compared to populations south of Geraldton (Figure S8). When only considering populations around PG, PG forests and crevices (PG1-2, PGCr1-3) cluster together with the historical Kalbarri samples and a few samples from Horrocks (HOR1-2) and Geraldton (GER1-2) (Fig. 5). Although, four individuals from PG crevices (PGCr1,3) cluster together with Horrocks, two drift specimens and one individual from PG forests (PG2). One drift individual was assigned to the Geraldton cluster (Fig. 5).

**Fig. 6:**
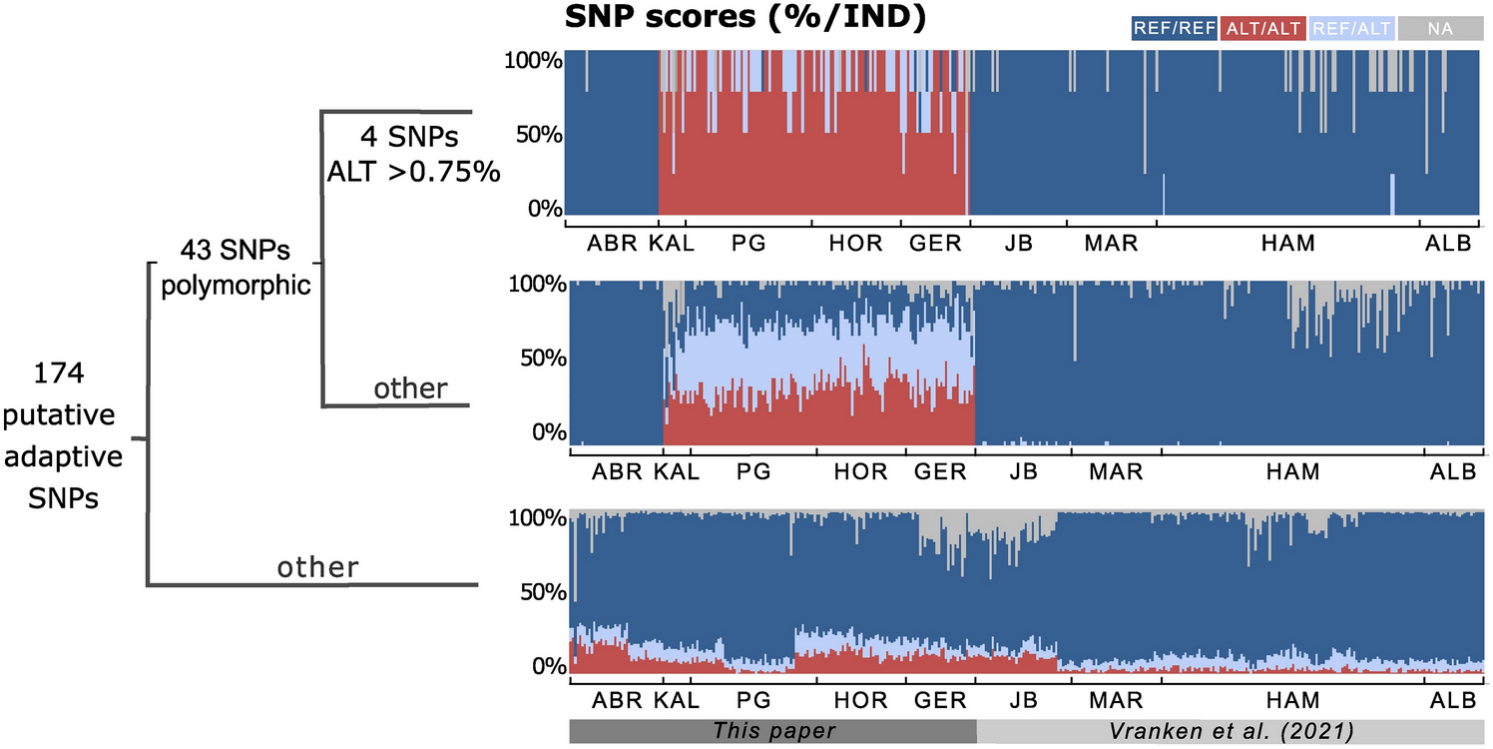
Proportion of genotype frequencies for 174 putative temperature-linked loci for all sampling sites discussed in this paper and other West Australian sampling sites discussed in Vranken et al. (2021) (Figure S8B). Loci are grouped based on the proportion of the alternative allele. 43 SNPs were only polymorphic in the broader PG region of which 4 SNPs the alternative allele frequency was > 75%. REF = reference, ALT = alternative and NA = missing data. ABR = Abrolhos, PG = Port Gregory, HOR = Horrocks, GER = Geraldton, JB = Jurien Bay, MAR = Marmion, HAM = Hamelin, ALB = Albany.

Remarkably, 43 SNPs (25%) showed allelic polymorphism uniquely present in the broader PG region (KAL, PG1-2, PGCr1-3, GER1-2, HOR1-2) compared to populations sampled poleward of Geraldton (Fig. 6). Of these, four SNPs showed almost full fixation for the alternative allele (> 0.75%) relative to other West Australian sites (which were monomorphic) (Fig. 6), potentially indicating a selective sweep event. Four SNPs that were uniquely polymorphic for the broader PG region (including one SNP showing almost full fixation for the alternative allele) were linked to fundamental cellular functions involved in sterol activity, signal transduction, Heat Shock Proteins (HSP) and N-acetyltransferase activity (Supplementary Table S7).

## Discussion

Extreme climatic events (ECE)s, including Marine heatwaves (MHWs), are expected to become more prevalent and severe throughout the twenty-first century^2,3,72^ with devastating impacts to ecosystems. Understanding the mechanisms behind resilience and recolonisation is crucial for managing and conserving populations into the future. Our genetic clustering and assignment analysis indicate that the newly discovered PG kelp populations (PG1-2) were most likely survivors from the 2011 MHW with some genetic enrichment from new migrants from surrounding coastal populations. In contrast, a newly discovered small kelp population residing in crevices appears to be immigrants from a mix of northern and southern populations, indicating multidirectional geneflow (migrant model sensu Slatkin^15^). This was reflected through higher genetic diversity, limited inbreeding and similar potentially adaptive diversity in all newly discovered populations, suggesting that the PG range edge populations likely have good adaptive capacity and resilience to climatic stress^73^.

Kelps and macroalgae are being impacted by extreme events worldwide^18,28^, often leading to local extinction, especially at lower latitudes where the chances of exceeding physiological temperature thresholds are higher^25,38,74,75^. The relationship between heatwave intensity and kelp mortality has been well demonstrated e.g.^26^. Based on satellite derived SST values, the 2011 MHW was more intense in Kalbarri (category IV^76^) than in PG (forests and crevices) (category III) (Fig. S1), which agrees with the extinction of kelps in Kalbarri and some survival in PG forests (PG1-2). Although MHW intensity for PG forests and PG crevices was similar, no survival was observed in PG crevices, which suggests that other factors may have driven susceptibility to the 2011 MHW. One explaining factor could be that local fine-scale temperature variability was not well captured by the SST, making SST an unreliable variable to estimate MHW impact on kelps, as has been shown for as has been shown for *Ericaria crinita* in the Mediterranean Sea^77^. Also, fine-scale differences in other abiotic variables such as wave exposure might play a role in mediating MHW susceptibility of kelp^78^.

Resilience of kelp forests will be affected by the initial impact of the heatwave. The stronger the impact, the more challenged recovery will be (~ Allee effects). But resilience is also dependent on interacting abiotic and biotic variables such as grazing, competition and propagule availability^28,75,79^, which can cause significant differences in resilience and recovery time among locations^10,75^. Ten years after the 2011 MHW, we observed significant differences among nearby sites, ranging from complete lack of recovery in Kalbarri^20^ to resilience through sporadic recolonisation at the PG crevices and resilience through a combination of survival and recolonisation at PG forests. After the 2011 MHW, kelp recovery in remnant sites was significantly inhibited by an influx of tropical herbivores^39^. Indeed, Zarco‐Perello, et al.^41^ found that herbivory pressure (mainly by *Siganus fuscescens* and *Kyphosus bigibbus*) is still high at the PG crevice sites (PGCr1-3) with exposed adult kelps losing up to 80% of biomass in 48 h due to herbivory, compared to 6% of biomass for protected kelps, sheltered in crevices. Spatial heterogeneity in herbivore grazing pressure might explain differences in kelp occurrence between PG forests, PG crevices and Kalbarri, although research specifically investigating herbivory at all these places would be needed to support this hypothesis.

Dispersal of propagules followed by effective geneflow via recruitment, survival and reproduction is crucial for population survival and recolonisation after local extinction events^80^, as shown, for instance, for corals^81^ and seaweeds^44,53,82^. Both PG forests and PG crevices revealed high admixture and connectivity with the surrounding kelp populations. In particular, the closest population, Horrocks, appears to act as an important source for the PG populations. Further, recovery from microscopic spore or gametophyte banks has also been suggested to initiate kelp recovery after climatic stress^50,51,83^, and may have contributed partially or mainly to rapid recovery of the surviving PG forests once environmental conditions improved after the MHW. Yet, In the case of the crevice populations, their persistence as a gametophyte bank is highly unlikely, because most kelp gametophytes persist for less than a year in the field^51^ and kelp sporophytes were only discovered 10 years after the heatwave despite regular visits to those sites.

In passively dispersing marine organisms like seaweeds, ocean currents play an important role in gene flow^84^. In Western Australia, the continental boundary Leeuwin current transports warm tropical water poleward along the complete coast of western Australia, and has been considered a key driver of unidirectional gene flow and population connectivity of *Ecklonia*^84^. However, our results indicate equatorward migration, potentially driven by inner shelf counter-currents such as the cool equatorward Capes Current, which is strongest when *Ecklonia* is fertile and produces zoospores^85^, or potential other mesoscale (e.g. eddies) or reef scale variables (e.g. wind). Kelp dispersal may occur via dispersal of zoospores, gametes or drifting reproductive sporophytes. The majority of *Ecklonia* zoospores are thought to mainly settle in a few metres from the parent plant (up to 20—40 m)^86–88^, as motility of kelp spores often decreases markedly within short short-timeframes^48^. Yet, passive dispersal of zoospores and dispersal of detached fertile sporophyte material may contribute to long distance dispersal^48,89^. Our sequencing results of healthy drift individuals confirm that sporophytes can travel distances of at least > 25 km^56^, potentially maintaining the capacity to disperse reproductive material. Indeed, this is the likely pathway by which kelps at PG crevices recolonised, as washed-up sporophytes are regularly observed on the sandy beaches near PG crevices (authors’ personal observation), likely dispersing from both Horrocks and PG forests. The lack of recolonisation at the historical range edge Kalbarri sites might be explained by connectivity that is too weak for reproductive propagules or drift individuals to disperse successfully from surviving forests to Kalbarri (> 40 km). Oceanographic or biophysical connectivity modelling would help clarify this hypothesis.

The 2011 MHW affected kelp performance by inducing stress and mortality, and reorganising entire ecological communities^24,38^. This mortality can reduce standing genetic diversity and potentially restrict future adaptive evolution^90^. Conversely, when mortality has arisen through selection, it can enhance future resilience to the same stressors that caused mortality^91–93^. Our findings indicate high overall genetic diversity and similar putative adaptive diversity of the recolonising kelp relative to surviving populations, suggesting good population fitness and likely adaptation to temperature stress. Although, it should be noted that there can be a time lag of multiple generations after a disturbance before the impact on genetic diversity becomes measurable^94^. Remarkably, the surviving populations show the same patterns of putative adaptation towards temperature as the extirpated Kalbarri samples. This suggests that the extirpated Kalbarri populations were likely similarly adapted to temperature stress but that the extreme temperature conditions (MHW category IV) exceeded physiological thresholds and that the surviving PG populations remain vulnerable to extreme temperature conditions (Fig. S1). Populations northwards of Geraldton, which exhibited the highest losses following the 2011 MHW (> 80% cover loss^24^), showed allelic polymorphism for 25% of the putative SNPs linked to temperature, including a candidate SNP (SNP ID: 2032_6) linked to a Heat Shock Protein 40 like protein, which could indicate a selection event. Moreover, four SNPs are almost fully fixed for the alternative allele, which could indicate a strong selective advantage that became almost fully fixed in the populations, i.e. a selective sweep^95^. To confirm whether these populations are more resistant to temperature stress, manipulative experiments would be needed to link performance with genetic signals. In particular, a good candidate for further research is one SNP that linked to potential N-acetyltransferase activity (SNP ID: 8241_124) which is anticipated to act as an important element coordinating metabolic, developmental and physiological response to abiotic stress-tolerance^96^. Thermal stress experiments on individuals with different alleles at these SNPs under putative selection could demonstrate causation. In addition, to provide empirical confirmation whether the heatwaves caused selection or any other genetic changes in the PG populations, analyses of temporal samples would be needed. But as extreme events are very hard to predict in time and space, “before” data is often not available^91,97^.

Our ddRAD-sequencing demonstrate resilience of kelp forests following a marine heatwave in PG, Western Australia. Resilience has been accomplished through a mix of survival through the heatwave and subsequent recruitment from surrounding areas following loss. We reveal likely small-scale difference in kelp resistance due to spatially variable abiotic (temperature) and biotic (herbivory) conditions that have led to this variable pattern of recovery. Population connectivity through drifting sporophytes is critical in facilitating resilience to ECE but zoospore dipersal and microscopic banks may also play a role in recovery in surviving populations (PG forests). We were able to identify the most important propagule source population (Horrocks) for the new range edge recovering populations in Port Gregory. Management strategies aiming to protect the recovering kelp in Port Gregory, will therefore be the most effective when also the kelp forests in Horrocks are protected. Moreover, any potential restoration efforts can be guided by natural scales of dispersal or potentially, knowledge of selection for thermal tolerance if assisted adaptation is required. Our results confirm that ECEs profoundly impact entire ecosystems and that resilience to ECEs can be complex and driven by small scale processes. Understanding these processes is crucial to estimate the impact of future ECEs and developing effective management, restoration and conservation strategies.

## Supplementary Information

Supplementary Information.

## Data Availability

Vcf-files were deposited on FigShare and are available at the following URL: 10.6084/m9.figshare.25524181.

## References

[1] Perkins-Kirkpatrick, S. & Lewis, S. Increasing trends in regional heatwaves. *Nat. Commun.* **11**, 1–8 (2020). 32620857 10.1038/s41467-020-16970-7 PMC7334217

[2] Frölicher, T. L., Fischer, E. M. & Gruber, N. Marine heatwaves under global warming. *Nature* **560**, 360–364 (2018). 30111788 10.1038/s41586-018-0383-9

[3] Oliver, E. C. et al. Projected marine heatwaves in the 21st century and the potential for ecological impact. *Front. Mar. Sci.* **6**, 734 (2019).

[4] Pohl, B., Macron, C. & Monerie, P.-A. Fewer rainy days and more extreme rainfall by the end of the century in Southern Africa. *Sci. Rep.* **7**, 1–7 (2017). 28406241 10.1038/srep46466 PMC5390289

[5] Russo, S., Marchese, A. F., Sillmann, J. & Immé, G. When will unusual heat waves become normal in a warming Africa?. *Environ. Res. Lett.* **11**, 054016 (2016).

[6] Harris, R. M. et al. Biological responses to the press and pulse of climate trends and extreme events. *Nat. Clim. Change* **8**, 579–587 (2018).

[7] Smith, K. E. et al. Biological Impacts of Marine Heatwaves. *Annu. Rev. Mar. Sci.* **15**, 119–145. 10.1146/annurev-marine-032122-121437 (2023). 10.1146/annurev-marine-032122-121437 35977411

[8] Maxwell, S. L. et al. Conservation implications of ecological responses to extreme weather and climate events. *Divers. Distrib.* **25**, 613–625. 10.1111/ddi.12878 (2019).

[9] Hodgson, D., McDonald, J. L. & Hosken, D. J. What do you mean, ‘resilient’?. *Trends Ecol. Evol.* **30**, 503–506 (2015). 26159084 10.1016/j.tree.2015.06.010

[10] Cavanaugh, K. C., Reed, D. C., Bell, T. W., Castorani, M. C. & Beas-Luna, R. Spatial variability in the resistance and resilience of giant kelp in southern and Baja California to a multiyear heatwave. *Front. Mar. Sci.* **6**, 413 (2019).

[11] Wernberg, T. et al. Decreasing resilience of kelp beds along a latitudinal temperature gradient: Potential implications for a warmer future. *Ecol. Lett.* **13**, 685–694 (2010). 20412279 10.1111/j.1461-0248.2010.01466.x

[12] Xuereb, A., D’Aloia, C. C., Andrello, M., Bernatchez, L. & Fortin, M.-J. Incorporating putatively neutral and adaptive genomic data into marine conservation planning. *Conservat. Biol.* **35**, 909–920. 10.1111/cobi.13609 (2021). 10.1111/cobi.13609 32785955

[13] Hughes, A. R., Inouye, B. D., Johnson, M. T., Underwood, N. & Vellend, M. Ecological consequences of genetic diversity. *Ecol. Lett.* **11**, 609–623 (2008). 18400018 10.1111/j.1461-0248.2008.01179.x

[14] Coleman, M. A. & Kelaher, B. P. Connectivity among fragmented populations of a habitat-forming alga, Phyllospora comosa (Phaeophyceae, Fucales) on an urbanised coast. *Mar. Ecol. Prog. Ser.* **381**, 63–70 (2009).

[15] Slatkin, M. Gene flow and genetic drift in a species subject to frequent local extinctions. *Theor. Popul. Biol.* **12**, 253–262 (1977). 601717 10.1016/0040-5809(77)90045-4

[16] Hobday, A. J. et al. A hierarchical approach to defining marine heatwaves. *Progress Oceanogr.* **141**, 227–238 (2016).

[17] Oliver, E. C. et al. Longer and more frequent marine heatwaves over the past century. *Nat. Commun.* **9**, 1–12 (2018). 29636482 10.1038/s41467-018-03732-9 PMC5893591

[18] Smale, D. A. Impacts of ocean warming on kelp forest ecosystems. *New Phytol.* **225**, 1447–1454. 10.1111/nph.16107 (2020). 31400287 10.1111/nph.16107

[19] Smale, D. A. et al. Marine heatwaves threaten global biodiversity and the provision of ecosystem services. *Nat. Clim. Change* **9**, 306–312 (2019).

[20] Smith, K. E. et al. Socioeconomic impacts of marine heatwaves: Global issues and opportunities. *Science* **374**, eabj3593 (2021). 34672757 10.1126/science.abj3593

[21] Moore, J. A. et al. Unprecedented mass bleaching and loss of coral across 12 of latitude in Western Australia in 2010–11. *PLoS One* **7**, e51807 (2012). 23284773 10.1371/journal.pone.0051807 PMC3524109

[22] Hughes, T. P. et al. Global warming and recurrent mass bleaching of corals. *Nature* **543**, 373 (2017). 28300113 10.1038/nature21707

[23] Kendrick, G. A. et al. A systematic review of how multiple stressors from an extreme event drove ecosystem-wide loss of resilience in an iconic seagrass community. *Front. Mar. Sci.* **6**, 455 (2019).

[24] Wernberg, T. et al. Climate-driven regime shift of a temperate marine ecosystem. *Science* **353**, 169–172. 10.1126/science.aad8745 (2016). 27387951 10.1126/science.aad8745

[25] Arafeh-Dalmau, N. et al. Extreme marine heatwaves alter kelp forest community near its equatorward distribution limit. *Front. Mar. Sci.* **6**, 499 (2019).

[26] Filbee-Dexter, K. et al. Marine heatwaves and the collapse of marginal North Atlantic kelp forests. *Sci. Rep.* **10**, 1–11 (2020). 32770015 10.1038/s41598-020-70273-x PMC7414212

[27] Thomsen, M. S. et al. Local extinction of bull kelp (Durvillaea spp.) due to a marine heatwave. *Front. Mar. Sci.* **6**, 84 (2019).

[28] Wernberg, T., Krumhansl, K., Filbee-Dexter, K. & Pedersen, M. F. In *World Seas: An Environmental Evaluation (Second Edition)* (ed. Sheppard, C.) 57–78 (Academic Press, 2019).

[29] Brown, C. J., Mellin, C., Edgar, G. J., Campbell, M. D. & Stuart-Smith, R. D. Direct and indirect effects of heatwaves on a coral reef fishery. *Glob. Change Biol.* **27**, 1214–1225 (2021). 10.1111/gcb.15472 33340216

[30] Brothers, C. & McClintock, J. The effects of climate-induced elevated seawater temperature on the covering behavior, righting response, and Aristotle’s lantern reflex of the sea urchin Lytechinus variegatus. *J. Exp. Mar. Biol. Ecol.* **467**, 33–38 (2015).

[31] Rovira, G. l. *et al.* When resilience is not enough: 2022 extreme marine heatwave threatens climatic refugia for a habitat-forming Mediterranean octocoral. *J. Anim. Ecol.*10.1111/1365-2656.14112. 10.1111/1365-2656.14112 PMC12326063 38867406

[32] Garrabou, J. et al. Mass mortality in Northwestern Mediterranean rocky benthic communities: Effects of the 2003 heat wave. *Glob. Change Biol.* **15**, 1090–1103. 10.1111/j.1365-2486.2008.01823.x (2009).

[33] Coleman, M. A. et al. Loss of a globally unique kelp forest from Oman. *Sci. Rep.* **12**, 5020. 10.1038/s41598-022-08264-3 (2022). 35322059 10.1038/s41598-022-08264-3 PMC8943203

[34] Wernberg, T. *Ecosystem Collapse and Climate Change* 325–343 (Springer, 2021).

[35] Pearce, A. F. & Feng, M. The rise and fall of the “marine heat wave” off Western Australia during the summer of 2010/2011. *J. Mar. Syst.* **111**, 139–156 (2013).

[36] Le Nohaïc, M. et al. Marine heatwave causes unprecedented regional mass bleaching of thermally resistant corals in northwestern Australia. *Sci. Rep.* **7**, 1–11 (2017). 29101362 10.1038/s41598-017-14794-y PMC5670227

[37] Smale, D. A. & Wernberg, T. Extreme climatic event drives range contraction of a habitat-forming species. *Proc. R. Soc. B* **280**, 20122829 (2013). 23325774 10.1098/rspb.2012.2829 PMC3574333

[38] Wernberg, T. et al. An extreme climatic event alters marine ecosystem structure in a global biodiversity hotspot. *Nat. Clim. Change* **3**, 78 (2013).

[39] Bennett, S., Wernberg, T., Harvey, E. S., Santana-Garcon, J. & Saunders, B. J. Tropical herbivores provide resilience to a climate-mediated phase shift on temperate reefs. *Ecol. Lett.* **18**, 714–723 (2015). 25994785 10.1111/ele.12450

[40] Bosch, N. E. et al. Persistent thermally driven shift in the functional trait structure of herbivorous fishes: Evidence of top-down control on the rebound potential of temperate seaweed forests?. *Glob. Change Biol.* **28**, 2296–2311 (2022). 10.1111/gcb.16070 34981602

[41] Zarco‐Perello, S., Bosch, N. E., Bennett, S., Vanderklift, M. A. & Wernberg, T. Persistence of tropical herbivores in temperate reefs constrains kelp resilience to cryptic habitats. *J. Ecol.* (2021).

[42] Proft, K. M., Jones, M. E., Johnson, C. N. & Burridge, C. P. Making the connection: Expanding the role of restoration genetics in restoring and evaluating connectivity. *Restor. Ecol.* **26**, 411–418 (2018).

[43] Coleman, M. A. & Goold, H. Harnessing synthetic biology for kelp forest conservation. *J. Phycol.* **55**, 745–751 (2019). 31152453 10.1111/jpy.12888

[44] Waters, J. M., Fraser, C. I. & Hewitt, G. M. Founder takes all: Density-dependent processes structure biodiversity. *Trends Ecol. Evol.* **28**, 78–85 (2013). 23000431 10.1016/j.tree.2012.08.024

[45] Cherry, J. L. Selection, subdivision and extinction and recolonization. *Genetics* **166**, 1105–1114 (2004). 15020490 10.1093/genetics/166.2.1105 PMC1470724

[46] McCauley, D. E. Genetic consequences of local population extinction and recolonization. *Trends Ecol. Evol.* **6**, 5–8 (1991). 21232411 10.1016/0169-5347(91)90139-O

[47] Pannell, J. R. & Charlesworth, B. Neutral genetic diversity in a metapopulation with recurrent local extinction and recolonization. *Evolution* **53**, 664–676 (1999). 28565620 10.1111/j.1558-5646.1999.tb05362.x

[48] Wernberg, T. et al. Biology and ecology of the globally significant kelp Ecklonia radiata. *Oceanogr. Mar. Biol. Annu. Rev.* **57**, 265–324 (2019).

[49] Edwards, M. S. It’s the little things: The role of microscopic life stages in maintaining kelp populations. *Front. Mar. Sci.* **9**, 871204 (2022).

[50] Ladah, L. B., Zertuche-González, J. A. & Hernández-Carmona, G. Giant kelp (Macrocystis pyrifera, Phaeophyceae) recruitment near its southern limit in Baja California after mass disappearance during ENSO 1997–1998. *J. Phycol.* **35**, 1106–1112 (1999).

[51] Veenhof, R. J. et al. *Oceanography and Marine Biology* 335–371 (CRC Press, 2022).

[52] Vranken, S., Scheben, A., Batley, J., Wernberg, T. & Coleman, M. A. Genomic consequences and selection efficacy in sympatric sexual versus asexual kelps. *Front. Mar. Sci.*10.3389/fmars.2022.921912 (2022).

[53] Peters, J. C., Waters, J. M., Dutoit, L. & Fraser, C. I. SNP analyses reveal a diverse pool of potential colonists to earthquake-uplifted coastlines. *Mol. Ecol.* **29**, 149–159. 10.1111/mec.15303 (2020). 31711270 10.1111/mec.15303

[54] Macaya, E. C. & Zuccarello, G. C. Genetic structure of the giant kelp Macrocystis pyrifera along the southeastern Pacific. *Mar. Ecol. Prog. Ser.* **420**, 103–112. 10.3354/meps08893 (2010).

[55] Fraser, C. I., Nikula, R., Spencer, H. G. & Waters, J. M. Kelp genes reveal effects of subantarctic sea ice during the Last Glacial Maximum. *Proc. Natl. Acad. Sci.* **106**, 3249–3253 (2009). 19204277 10.1073/pnas.0810635106 PMC2651250

[56] Kirkman, H. & Kendrick, G. A. Ecological significance and commercial harvesting of drifting and beach-cast macro-algae and seagrasses in Australia: A review. *J. Appl. Phycol.* **9**, 311–326 (1997).

[57] Vranken, S. et al. Genotype-environment mismatch of kelp forests under climate change. *Mol. Ecol.* **30**, 3730–3746 (2021). 34018645 10.1111/mec.15993

[58] Sundqvist, L., Keenan, K., Zackrisson, M., Prodöhl, P. & Kleinhans, D. Directional genetic differentiation and relative migration. *Ecol. Evol.* **6**, 3461–3475. 10.1002/ece3.2096 (2016). 27127613 10.1002/ece3.2096 PMC4842207

[59] Catchen, J., Hohenlohe, P. A., Bassham, S., Amores, A. & Cresko, W. A. Stacks: An analysis tool set for population genomics. *Mol. Ecol.* **22**, 3124–3140 (2013). 23701397 10.1111/mec.12354 PMC3936987

[60] Paris, J. R., Stevens, J. R. & Catchen, J. M. Lost in parameter space: A road map for stacks. *Methods Ecol. Evol.* **8**, 1360–1373 (2017).

[61] Danecek, P. et al. The variant call format and VCFtools. *Bioinformatics* **27**, 2156–2158 (2011). 21653522 10.1093/bioinformatics/btr330 PMC3137218

[62] Puritz, J. B., Hollenbeck, C. M. & Gold, J. R. dDocent: A RADseq, variant-calling pipeline designed for population genomics of non-model organisms. *PeerJ* **2**, e431 (2014). 24949246 10.7717/peerj.431 PMC4060032

[63] Jombart, T. adegenet: A R package for the multivariate analysis of genetic markers. *Bioinformatics* **24**, 1403–1405 (2008). 18397895 10.1093/bioinformatics/btn129

[64] Frichot, E. & François, O. LEA: An R package for landscape and ecological association studies. *Methods Ecol. Evol.* **6**, 925–929 (2015).

[65] Jombart, T. & Collins, C. (Imperial College London, London, 2017).

[66] Dias, P. C. Sources and sinks in population biology. *Trends Ecol. Evol.* **11**, 326–330 (1996). 21237863 10.1016/0169-5347(96)10037-9

[67] Keenan, K., McGinnity, P., Cross, T. F., Crozier, W. W. & Prodöhl, P. A. diveRsity: An R package for the estimation and exploration of population genetics parameters and their associated errors. *Methods Ecol. Evol.* **4**, 782–788. 10.1111/2041-210X.12067 (2013).

[68] Chen, K. Y. et al. assign POP: An r package for population assignment using genetic, non-genetic, or integrated data in a machine-learning framework. *Methods Ecol. Evol.* **9**, 439–446 (2018).

[69] Pembleton, L. W., Cogan, N. O. & Forster, J. W. StAMPP: An R package for calculation of genetic differentiation and structure of mixed-ploidy level populations. *Mol. Ecol. Resour.* **13**, 946–952 (2013). 23738873 10.1111/1755-0998.12129

[70] Schmidt, T. L., Jasper, M. E., Weeks, A. R. & Hoffmann, A. A. Unbiased population heterozygosity estimates from genome-wide sequence data. *Methods Ecol. Evol.* **12**, 1888–1898 (2021).

[71] Adamack, A. T. & Gruber, B. PopGenReport: Simplifying basic population genetic analyses in R. *Methods Ecol. Evol.* **5**, 384–387 (2014).

[72] IPCC. Climate change and land: an IPCC special report on climate change, desertification, land degradation, sustainable land management, food security, and greenhouse gas fluxes in terrestrial ecosystems. (Geneva, 2019).

[73] Wernberg, T. et al. Genetic diversity and kelp forest vulnerability to climatic stress. *Sci. Rep.* **8**, 1851 (2018). 29382916 10.1038/s41598-018-20009-9 PMC5790012

[74] Vergés, A. et al. The tropicalization of temperate marine ecosystems: Climate-mediated changes in herbivory and community phase shifts. *Proc. R. Soc. B* **281**, 20140846. 10.1098/rspb.2014.0846 (2014). 25009065 10.1098/rspb.2014.0846 PMC4100510

[75] Edwards, M. S. & Estes, J. A. Catastrophe, recovery and range limitation in NE Pacific kelp forests: A large-scale perspective. *Mar. Ecol. Prog. Ser.* **320**, 79–87 (2006).

[76] Hobday, A. J. et al. Categorizing and naming marine heatwaves. *Oceanography* **31**, 162–173 (2018).

[77] Verdura, J. et al. Local-scale climatic refugia offer sanctuary for a habitat-forming species during a marine heatwave. *J. Ecol.* **109**, 1758–1773 (2021).

[78] Starko, S. et al. Environmental heterogeneity mediates scale-dependent declines in kelp diversity on intertidal rocky shores. *PLoS One* **14**, e0213191 (2019). 30913219 10.1371/journal.pone.0213191 PMC6435185

[79] Edwards, M. & Hernandez-Carmona, G. Delayed recovery of giant kelp near its southern range limit in the North Pacific following El Niño. *Mar. Biol.* **147**, 273–279 (2005).

[80] Hanski, I. Metapopulation dynamics. *Nature* **396**, 41–49. 10.1038/23876 (1998).

[81] Underwood, J. N., Smith, L. D., van Oppen, M. J. & Gilmour, J. P. Multiple scales of genetic connectivity in a brooding coral on isolated reefs following catastrophic bleaching. *Mol. Ecol.* **16**, 771–784 (2007). 17284210 10.1111/j.1365-294X.2006.03187.x

[82] Becheler, R. et al. After a catastrophe, a little bit of sex is better than nothing: Genetic consequences of a major earthquake on asexual and sexual populations. *Evol. Appl.* **13**, 2086–2100 (2020). 32908606 10.1111/eva.12967 PMC7463374

[83] Schoenrock, K. M., McHugh, T. A. & Krueger-Hadfield, S. A. Revisiting the ‘bank of microscopic forms’ in macroalgal-dominated ecosystems. *J. Phycol.* **57**, 14–29 (2021). 33135166 10.1111/jpy.13092

[84] Coleman, M. A. et al. Variation in the strength of continental boundary currents determines continent-wide connectivity in kelp. *J. Ecol.* **99**, 1026–1032. 10.1111/j.1365-2745.2011.01822.x (2011).

[85] Pattiaratchi, C. Surface and sub-surface circulation and water masses off Western Australia. *Bull. Austr. Meteorol. Oceanogr. Soc.* **19**, 95–104 (2006).

[86] Choi, C. G., Serisawa, Y., Ohno, M. & Sohn, C.-H. Contruction of artificial seaweed beds; using the spore bag method. *Algae* **15**, 179–182 (2000).

[87] Serisawa, Y. et al. Marine aforestation of Ecklonia cava by using a spore bag method at an ISOYAKE area in Tosa Bay, southern Japan. *Jap. J. Phycol.* **53**, 19–24 (2005).

[88] Suzuki, H. et al. Local versus regional patterns in zoospore dispersal of the kelp Eisenia bicyclis (Laminariales, Phaeophyceae). *Phycol. Res.* **72**, 238–248. 10.1111/pre.12563 (2024).

[89] Choi, D. M., Ko, Y. W., Kang, R.-S. & Kim, J. H. Morphological and genetic variability among Ecklonia cava (Laminariales, Phaeophyceae) populations in Korea. *Algae* **30**, 89–101 (2015).

[90] Gurgel, C. F. D., Camacho, O., Minne, A. J., Wernberg, T. & Coleman, M. A. Marine heatwave drives cryptic loss of genetic diversity in underwater forests. *Curr. Biol.* **30**, 1199–1206 (2020). 32109397 10.1016/j.cub.2020.01.051

[91] Grant, P. R. et al. Evolution caused by extreme events. *Philos. Trans. R. Soc. B Biol. Sci.* **372**, 20160146 (2017). 10.1098/rstb.2016.0146 PMC5434096 28483875

[92] Coleman, M. A. & Wernberg, T. The silver lining of extreme events. *Trends Ecol. Evol.*10.1016/j.tree.2020.08.013 (2020). 32958366 10.1016/j.tree.2020.08.013

[93] Coleman, M. A. & Wernberg, T. A glass half full: Solutions-oriented management under climate change. *Trends Ecol. Evol.* **S0169–5347**(0121), 00060–00064 (2021). 10.1016/j.tree.2021.02.009 33715918

[94] Dussex, N., Morales, H. E., Grossen, C., Dalén, L. & van Oosterhout, C. Purging and accumulation of genetic load in conservation. *Trends Ecol. Evol.*10.1016/j.tree.2023.05.008 (2023). 37344276 10.1016/j.tree.2023.05.008

[95] Weigand, H. & Leese, F. Detecting signatures of positive selection in non-model species using genomic data. *Zool. J. Linnean Soc.* **184**, 528–583. 10.1093/zoolinnean/zly007 (2018).

[96] Gibbs, D. J. Emerging functions for N-terminal protein acetylation in plants. *Trends Plant Sci.* **20**, 599–601 (2015). 26319188 10.1016/j.tplants.2015.08.008 PMC4601045

[97] Coleman, M. A., Minne, A. J. P., Vranken, S. & Wernberg, T. Genetic tropicalisation following a marine heatwave. *Sci. Rep.* **10**, 1–11 (2020). 32728196 10.1038/s41598-020-69665-w PMC7391769

