# Supplementary material for: Connectivity enhances resilience of marine forests after an extreme event
Source: Sci Rep. 2025 Feb 11;15:5019. doi: 10.1038/s41598-025-87449-y (PMC11814082; doi:10.1038/s41598-025-87449-y)
Supplement: Supplementary file 1 — Supplementary Information. [file 41598_2025_87449_MOESM1_ESM.docx]

**Supplementary Information**

**Relict population recovers from extreme event: genomic insights from a marine forest**

**Vranken Sofie^1,2,3^*, Wernberg Thomas^1,2,4^, Scheben Armin^3,5^, Pessarrodona Albert^1,2^, Jacqueline Batley^2^, Coleman Melinda Ann^2,7,8^***

^1^UWA Oceans Institute, 24 Fairway, Crawley, WA 6009, Australia.

^2^School of Biological Sciences, The University of Western Australia, 35 Stirling Highway, Crawley, WA 6009, Australia.

^3^Phycology Research group, Ghent University, Krijgslaan 281 S8, 9000 Gent, Belgium

^4^Institute of Marine Research, Nye Flødevigveien 20, 4817 His, Norway.

^5^Simons Center for Quantitative Biology, Cold Spring Harbor Laboratory, Cold Spring Harbor, New York, NY, 11724, USA.

^6^New South Wales Department of Primary Industries, Orange Agricultural Institute, 1447 Forrest Road, Orange, NSW 2800, Australia.

^7^New South Wales Fisheries, National Marine Science Centre, 2 Bay Drive, Coffs Harbour, NSW 2450, Australia.

^8^Southern Cross University, National Marine Science Centre, 2 Bay Drive, Coffs Harbour, NSW 2450, Australia.

*corresponding authors: [sofie.vranken@ugent.be](mailto:sofiemvranken@gmail.com), [melinda.coleman@dpi.nsw.gov.au](mailto:melinda.coleman@dpi.nsw.gov.au)

n

**Table of Contents:**

| **Figure S1** Sea surface temperature to characterise the 2011 marine heatwave. | Page 3 |
| --- | --- |
| **Figure S2** Clustering inferred with DAPC and sNMF for *Ecklonia radiata* for the main dataset including the Houtman Abrolhos Islands. | Page 4 |
| **Figure S3** Figure S3 Clustering inferred with DAPC and sNMF for *Ecklonia radiata* for the main dataset (6133 SNPs). | Page 5 |
| **Figure S4** Clustering inferred with DAPC and sNMF for Ecklonia radiata for the Kalbarri dataset (663 SNPs). | Page 5 |
| **Figure S5** Pairwise *F_S_*_T_ estimates among all sampling sites including the Abrolhos for the main dataset (6133 SNPs). | Page 6 |
| **Figure S6** Pairwise *F_ST_* estimates between genetic clusters as identified by DAPC for K=4 using the main dataset (6133 SNPs). | Page 6 |
| **Figure S7** Pairwise *F_ST_* estimates among all sampling sites using the dataset including the historical Kalbarri samples(663 SNPs), and in- (A) and excluding the Abrolhos (B). Sampling sites are ordered from north to south. | Page 7 |
| **Figure S8** DAPC scatter plot based on putative temperature-linked loci (174 SNPs) for individuals sampled along the complete coast of Western Australia taken from Vranken et al (2021). | Page 7 |
| **Table S1** Posterior assignment probabilities of individuals sampled at Port Gregory Crevices (PGCr1-3) to existing genetic clusters within the coastal populations (PG1, PG2, HOR1, HOR2, GER1) using the main dataset (*N*= 6133 SNPs) and DAPC analysis. | Page 9 |
| **Table S2**  Mean self-assignment rates across Monte-Carlo cross-validation tests for potential origin populations (PG1, PG2, HOR1, HOR2, GER1) using the main dataset | Page 9 |
| **Table S3** Posterior assignment probabilities of individuals sampled at the Porth Gregory forests (PG1-2) and Port Gregory Crevices (PGCr1-3) to the coastal populations (KAL, HOR1, HOR2, GER1) using the Kalbarri dataset (N= 663 SNPs). | Page 10 |
| **Table S4** Mean self-assignment rates across Monte-Carlo cross-validation tests for potential origin populations (KAL, HOR1, HOR2, GER1) using the Kalbarri dataset (N= 663 SNPs). | Page 11 |
| **Table S5** Wilcoxon test of expected Heterozygosity among sampling sites. | Page 12 |
| **Table S6** Genetic diversity metrics for genetic clusters as identified by DAPC at K=4. | Page 12 |
| **Table S7** Table S4 Annotation for *E. radiata* gene models containing candidate SNPs linked to temperature variables and showing unique allelic polymorphism in the broader Port Gregory area. | Page 13 |

**

*Figure S1 Sea surface temperature to characterise the 2011 marine heatwave across all locations visualised with heatwaveR package (Schlegel & Smit, 2018). Black line represents temperature, grey line represents climatology, and green lines represent upper and lower thermal event thresholds. Colours indicate the category of the marine heatwave (1-3) (yellow-red) according to Hobday et al. (2018) and percentage refers to the estimated forest loss after the 2011 marine heatwave (Wernberg et al. 2016).*

**
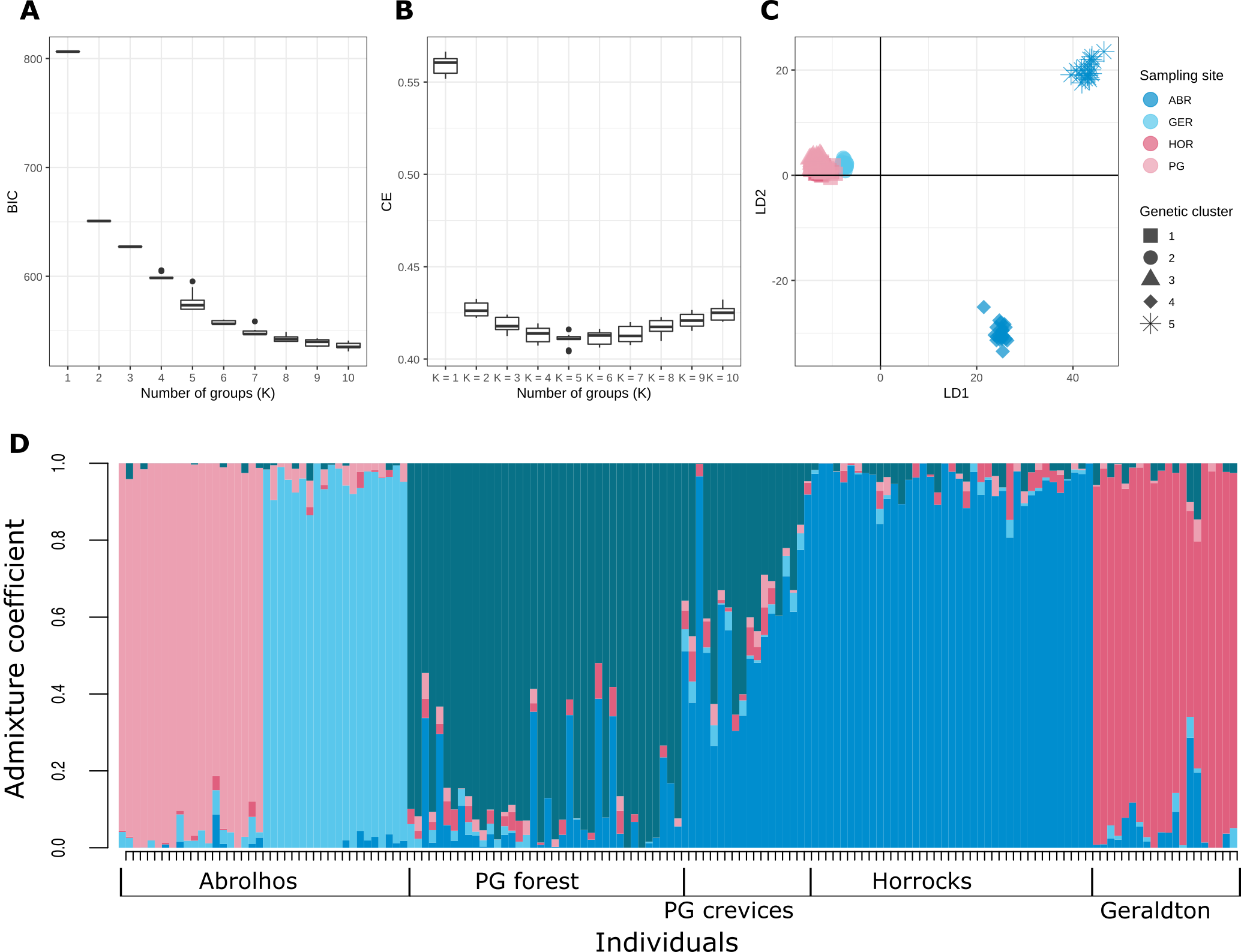
**

Figure S2 Clustering inferred with DAPC and sNMF for Ecklonia radiata for the main dataset including the Houtman Abrolhos Islands (6133 SNPs); (A) Bayesian information criteria (BIC) from sNMF analysis and (B) Cross Entropy from DAPC analysis for K = 1- 10; (c) DAPC scatter plot for K = 5, only the first 2 discriminant functions are shown; (D) sNMF admixture coefficients for K = 3 per sampling site, every bar represents one sampled individual with every colour representing the membership proportion of each cluster.

*Figure S3 Clustering inferred with DAPC and sNMF for Ecklonia radiata for the main dataset (6133 SNPs); (A) Bayesian information criteria (BIC) from DAPC analysis and (B) Cross Entropy from sNMF analysis for K = 1- 10.*

*Figure S4 Clustering inferred with DAPC and sNMF for Ecklonia radiata for the Kalbarri dataset (663 SNPs); (A) Bayesian information criteria (BIC) from DAPC analysis and Cross Entropy (CE) from sNMF analysis for K = 1- 10; (B) DAPC scatter plot for K = 3, only the first 2 discriminant functions are shown.*

*Figure S5* *Pairwise F_ST_ estimates among all sampling sites including the Abrolhos. Sampling sites are ordered from north to south using the main dataset (6133 SNPs).*

**

*Figure S6* *Pairwise F_ST_ estimates among genetic clusters as identified by DAPC for K=4 using the main dataset (6133 SNPs).*


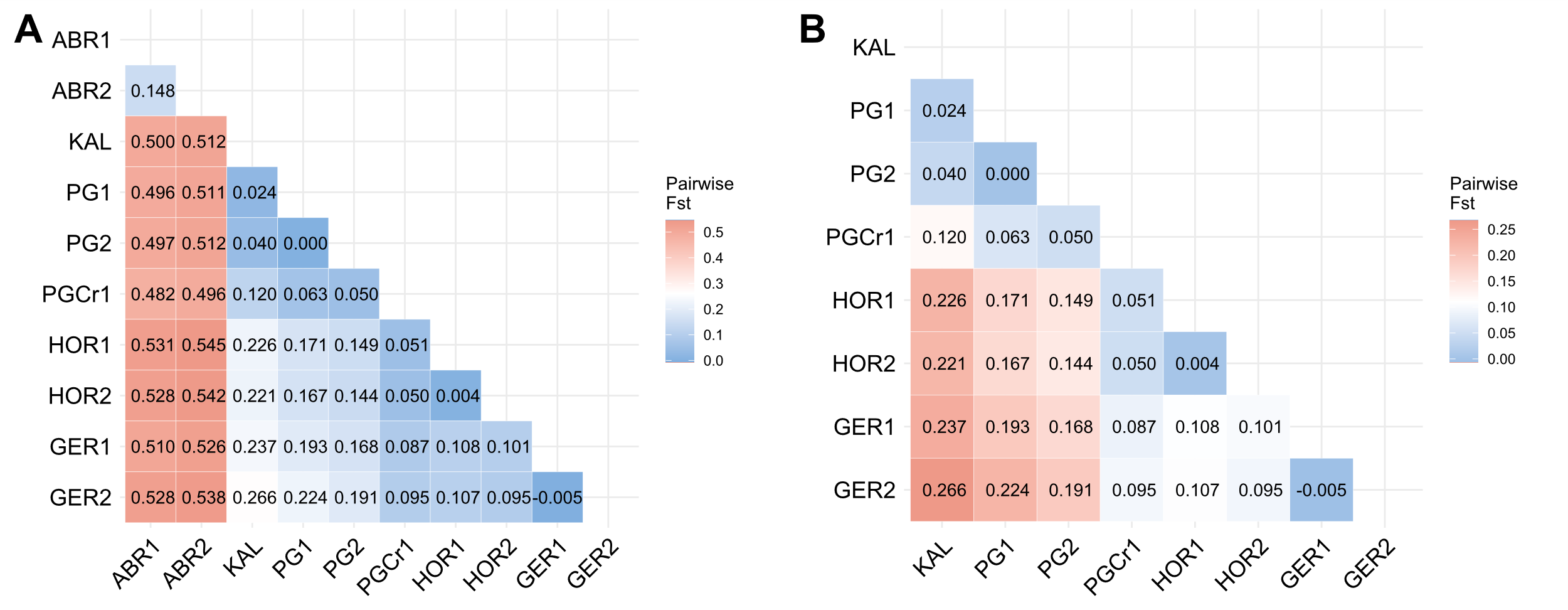


*Figure S7* *Pairwise F_ST_ estimates among all sampling sites including the Abrolhos (A) and excluding the Abrolhos (B) using the dataset including the historical Kalbarri samples(663 SNPs). Sampling sites are ordered from north to south.*

**A B**


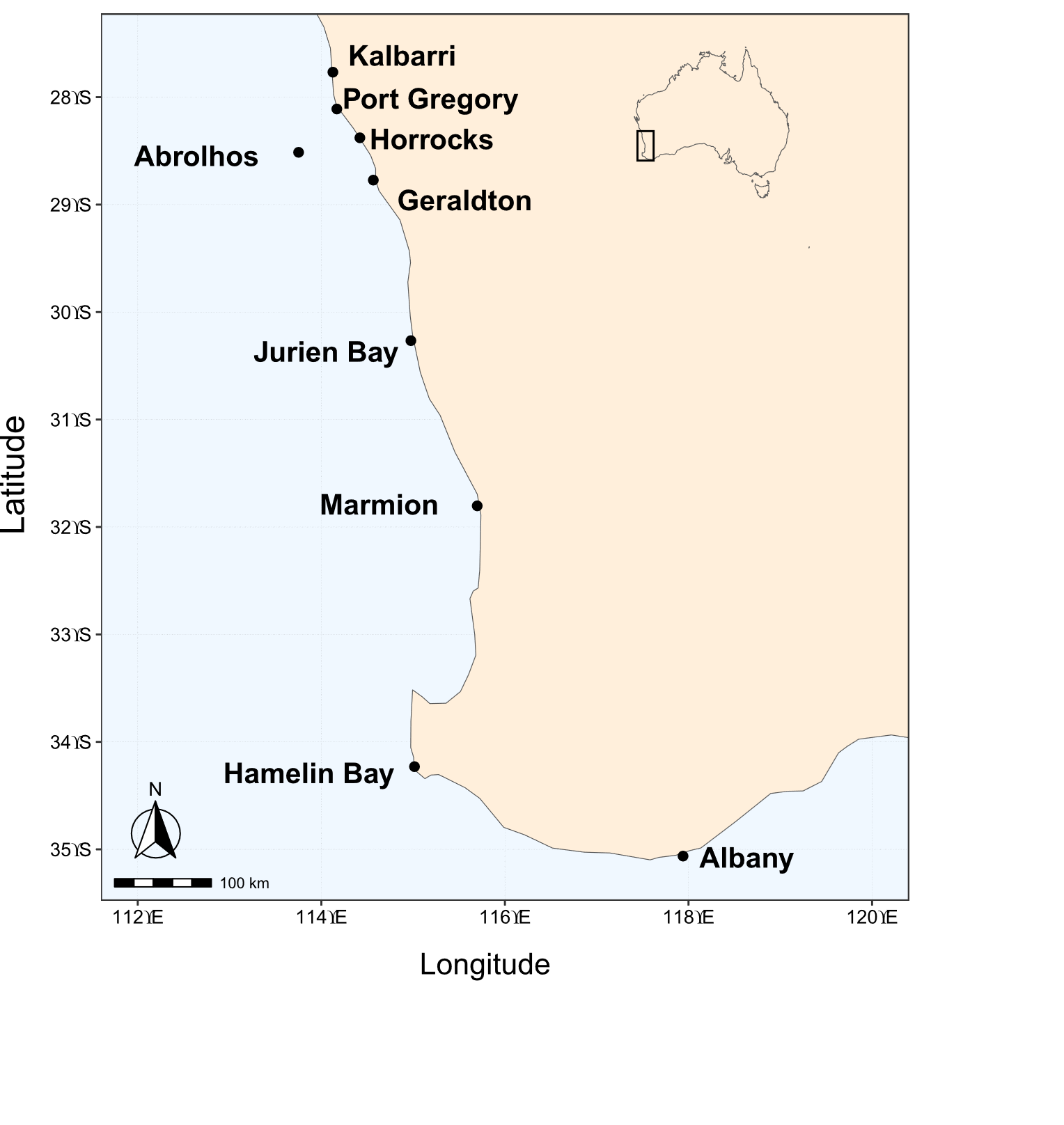


*Figure S8 DAPC scatter plot based on putative temperature-linked loci (174 SNPs) (A) for individuals sampled along the complete coast of Western Australia taken from Vranken et al (2021) (B). Only the 2 first discriminant functions are shown.*

*Table S1 Posterior assignment probabilities of individuals sampled at Port Gregory Crevices (PGCr1-3) to existing genetic clusters within the coastal populations (PG1, PG2, HOR1, HOR2, GER1) using the main dataset (N= 6133 SNPs) and DAPC analysis. Bold values correspond to successful assignments with probabilities equal to or higher than 0.95.*

| Individual | Collected at | ------------------------ Assigned to ------------------------ | | | | |
| --- | --- | --- | --- | --- | --- | --- |
|  |  | GER1 | HOR1 | HOR2 | PG1 | PG2 |
| PG121CI | PGCr1 | 0 | 0 | 0 | 0 | **1** |
| PG122CI | PGCr1 | 0 | 0 | 0 | 0 | **1** |
| PG123CI | PGCr1 | 0 | 0 | 0 | 0 | **1** |
| PG124CI | PGCr1 | 0 | 0 | 0 | 0 | **1** |
| PG125CI | PGCr1 | 0 | 0 | 0 | 0 | **1** |
| PG126CI | PGCr1 | 0 | 0 | 0 | 0 | **1** |
| PG127CI | PGCr1 | 0 | 0 | 0 | 0 | **1** |
| PG128CI | PGCr1 | 0 | 0 | 0 | 0 | **1** |
| PG130CI | PGCr1 | 0 | 0 | 0 | 0 | **1** |
| PG131CI | PGCr1 | 0 | 0 | 0 | 0 | **1** |
| PG132CI | PG drift | 0 | 0 | 0 | 0 | **1** |
| PG136CI | PGCr3 | 0 | 0 | 0 | 0 | **1** |
| PG137CI | PG drift | 0 | 0 | 0 | 0 | **1** |
| PG138CI | PG drift | 0 | 0 | 0 | 0 | **1** |
| PG141CI | PGCr2 | 0 | 0 | 0 | 0 | **1** |
| PG142CI | PGCr2 | 0 | 0 | 0 | 0 | **1** |
| PG143CI | PG drift | 0 | 0 | 0 | 0 | **1** |

*Table S2 Mean self-assignment rates across Monte-Carlo cross-validation tests for potential origin populations (PG1, PG2, HOR1, HOR2, GER1) using the main dataset (N= 6133 SNPs)*

|  | GER1 | HOR1 | HOR2 | PG1 | PG2 |
| --- | --- | --- | --- | --- | --- |
| GER1 | 1 | 0 | 0 | 0 | 0 |
| HOR1 | 0 | 1 | 0 | 0 | 0 |
| HOR2 | 0 | 0 | 1 | 0 | 0 |
| PG1 | 0 | 0 | 0 | 1 | 0 |
| PG2 | 0 | 0 | 0 | 0 | 1 |

*Table S3 Posterior assignment probabilities of individuals sampled at the Porth Gregory forests (PG1-2) and Port Gregory Crevices (PGCr1-3) to the coastal populations (KAL, HOR1, HOR2, GER1) using the Kalbarri dataset (N= 663 SNPs). Bold values correspond to successful assignments with probabilities equal to or higher than 0.95.*

| Individual | Collected at | --------------- Assigned to --------------- | | | | |
| --- | --- | --- | --- | --- | --- | --- |
|  |  | GER1 | GER2 | HOR1 | HOR2 | KAL |
| PG132CI | PG drift | 0 | 0 | **1** | 0 | 0 |
| PG136CI | PGCr3 | 0 | 0 | 0 | **1** | 0 |
| PG137CI | PG drift | 0 | 0 | **1** | 0 | 0 |
| PG138CI | PG drift | 0 | 0 | **0.99** | 0 | 0.01 |
| PG141CI | PGCr2 | 0 | 0 | 0.03 | 0 | **0.97** |
| PG142CI | PGCr2 | 0 | 0 | **0.95** | 0 | 0.05 |
| PG143CI | PG drift | 0 | 0 | 0.47 | 0.53 | 0 |
| PG121CI | PGCr1 | 0 | 0 | 0.84 | 0.16 | 0 |
| PG122CI | PGCr1 | 0 | 0 | **1** | 0 | 0 |
| PG123CI | PGCr1 | 0 | 0 | **0.96** | 0.04 | 0 |
| PG124CI | PGCr1 | 0 | 0 | 0.05 | **0.95** | 0 |
| PG125CI | PGCr1 | 0 | 0 | 0.44 | 0.56 | 0 |
| PG126CI | PGCr1 | 0 | 0 | **1** | 0 | 0 |
| PG127CI | PGCr1 | 0 | 0 | 0.03 | **0.97** | 0 |
| PG128CI | PGCr1 | 0 | 0 | 0 | **1** | 0 |
| PG130CI | PGCr1 | 0 | 0 | 0.01 | 0 | **0.99** |
| PG131CI | PGCr1 | 0 | 0 | 0.79 | 0.21 | 0 |
| PG31CI | PG2 | 0 | 0 | 0.51 | 0.48 | 0.01 |
| PG32CI | PG2 | 0 | 0 | 0.53 | 0.46 | 0.01 |
| PG33CI | PG2 | 0 | 0 | 0.01 | 0 | **0.98** |
| PG34CI | PG2 | 0 | 0 | 0.85 | 0.15 | 0 |
| PG35CI | PG2 | 0 | 0 | 0 | 1 | 0 |
| PG36CI | PG2 | 0 | 0 | 0.14 | 0.46 | 0.4 |
| PG37CI | PG2 | 0 | 0 | 0.68 | 0.12 | 0.19 |
| PG38CI | PG2 | 0 | 0 | 0.29 | 0.02 | 0.69 |
| PG40CI | PG2 | 0 | 0 | 0.94 | 0.06 | 0 |
| PG41CI | PG2 | 0 | 0 | 0.74 | 0.03 | 0.23 |
| PG42CI | PG2 | 0 | 0 | 0.03 | **0.97** | 0 |
| PG43CI | PG2 | 0 | 0 | 0.04 | 0.01 | **0.95** |
| PG44CI | PG2 | 0 | 0 | 0.01 | 0 | **0.99** |
| PG45CI | PG2 | 0 | 0 | 0.09 | 0.84 | 0.07 |
| PG46CI | PG2 | 0 | 0 | **0.96** | 0.03 | 0.02 |
| PG47CI | PG2 | 0 | 0 | 0 | 0 | 1 |
| PG48CI | PG2 | 0 | 0 | 0.38 | 0.48 | 0.14 |
| PG49CI | PG2 | 0 | 0 | 0.14 | 0.86 | 0 |
| PG51CI | PG2 | 0 | 0 | **0.99** | 0.01 | 0 |
| PG53CI | PG2 | 0 | 0 | **0.96** | 0.03 | 0.01 |
| PG10CI | PG1 | 0 | 0 | 0 | 0 | **1** |
| PG11CI | PG1 | 0 | 0 | 0 | 0 | **1** |
| PG12CI | PG1 | 0 | 0 | **1** | 0 | 0 |
| PG13CI | PG1 | 0 | 0 | 0 | 0 | **1** |
| PG14CI | PG1 | 0 | 0 | **1** | 0 | 0 |
| PG15CI | PG1 | 0 | 0 | 0.1 | 0 | **0.9** |
| PG16CI | PG1 | 0 | 0 | 0 | 0 | **1** |
| PG18CI | PG1 | 0 | 0 | 0.03 | 0 | **0.97** |
| PG20CI | PG1 | 0 | 0 | 0.01 | 0 | **0.99** |
| PG21CI | PG1 | 0 | 0 | 0 | 0 | **1** |
| PG22CI | PG1 | 0 | 0 | 0 | 0 | **1** |
| PG23CI | PG1 | 0 | 0 | **0.98** | 0 | 0.02 |
| PG24CI | PG1 | 0 | 0 | 0.82 | 0 | 0.18 |
| PG26CI | PG1 | 0 | 0 | 0.06 | 0.01 | 0.93 |
| PG2CI | PG1 | 0 | 0 | **1** | 0 | 0 |
| PG6CI | PG1 | 0 | 0 | 0 | 0 | **1** |
| PG7CI | PG1 | 0 | 0 | 0 | 0 | **1** |
| PG8CI | PG1 | 0 | 0 | 0.52 | 0 | 0.48 |

*Table S4 Mean self-assignment rates across Monte-Carlo cross-validation tests for potential origin populations (KAL, HOR1, HOR2, GER1) using the Kalbarri dataset (N= 663 SNPs).*

|  | GER1 | GER2 | HOR1 | HOR2 | KAL |
| --- | --- | --- | --- | --- | --- |
| GER1 | 1 | 0 | 0 | 0 | 0 |
| GER2 | 0 | 0.99 | 0 | 0 | 0 |
| HOR1 | 0 | 0 | 0.88 | 0.11 | 0 |
| HOR2 | 0 | 0.01 | 0.12 | 0.88 | 0 |
| KAL | 0 | 0.02 | 0.04 | 0.17 | 0.77 |

***Table S5:*** *P-values of the pairwise Wilcoxon test after correction with the bonferonni method to test for differences in levels of expected Heterozygosity (H_e_) among sampling sites. Only sampling sites with N>10 are included.*

|  | **PG1** | **PG2** | **PGCr1** | **HOR1** | **HOR2** |
| --- | --- | --- | --- | --- | --- |
| **PG2** | 1.00 | - | - | - | - |
| **PGCr1** | *0.01* | *<0.01* | - | - | - |
| **HOR1** | *<0.01* | *<0.01* | 1.00 | - | - |
| **HOR2** | *<0.01* | *<0.01* | 1.00 | 1.00 | - |
| **GER2** | *0.01* | *<0.01* | 1.00 | 1.00 | 1.00 |

***Table S6:*** *Genetic diversity metrics for genetic clusters as identified by DAPC at K=4, using the main dataset (N = 6133 SNPs)****.*** *Number of individuals genotyped (N), number of private alleles (N_P_), percentage of polymorphic loci (% Loci), observed heterozygosity (H_O_), Expected heterozygosity (H_E_), nucleotide diversity (π), and inbreeding coefficient (F_IS_)*

| **ID** | **N** | **N_P_** | **% Loci** | **H_O_** | **H_E_** | **π** | **F_IS_ ± SE** |
| --- | --- | --- | --- | --- | --- | --- | --- |
| ABR1 | 20 | 193 | 33.594 | 0.108 | 0.107 | 0.110 | 0.006 ± 0.016 |
| ABR2 | 20 | 215 | 34.477 | 0.105 | 0.107 | 0.110 | 0.019 ± 0.017 |
| PG forest cluster | 34 | 68 | 63.419 | 0.168 | 0.166 | 0.171 | 0.010 ± 0.110 |
| PG crevice cluster | 20 | 19 | 59.597 | 0.164 | 0.163 | 0.169 | 0.015 ± 0.049 |
| HOR cluster | 41 | 63 | 62.990 | 0.146 | 0.149 | 0.153 | 0.029 ± 0.110 |
| GER cluster | 20 | 138 | 56.983 | 0.163 | 0.157 | 0.161 | 0.004 ± 0.022 |

*Table S7 Annotation for E. radiata gene models containing candidate SNPs linked to temperature variables and showing unique allelic polymorphism in the broader Port Gregory area (compared to populations sampled more south of Geraldton along the West Australian coast). Hypothetical and conserved unknown proteins are not included. We provide SNP ID, annotation, molecular or biological GO terms and functions as described in literature and whether a trend of a putative selective sweep has been detected.*

| **SNP ID** | **Annotation** | **Function** | **Putative selective sweep** |
| --- | --- | --- | --- |
| 19176_33 | similar to transmembrane protein 97 [Ectocarpus siliculosus] | fundamental cellular function, plays a role in the cellular sterol levels (Sanchez-Pulido & Ponting, 2014) |  |
| 2032_6 | Heat shock protein 40 like protein [Ectocarpus siliculosus] | interacts with HSP70, HSP70 is involved in tolerance to heat stress (Park et al., 2012) |  |
| 48140_57 | Tyrosine kinase specific for activated (GTP-bound) p21cdc42Hs/ leucine rich repeat protein [Ectocarpus siliculosus] | ATP binding, protein kinase, signal transduction mechanisms (GO term) |  |
| 8241_124 | ribosomal-protein-alanine acetyltransferase-like protein [Ectocarpus siliculosus] | N-acetyltransferase activity (GO term) | Yes |
